# Genomic population structure, antimicrobial susceptibility, and clinical features of *Mycobacterium xenopi* isolates, Frankfurt, Germany, 1995–2020

**DOI:** 10.1128/jcm.01511-25

**Published:** 2026-02-09

**Authors:** Margo Diricks, Lisa Marschall, Teodora Biciusca, Ann-Sophie Zielbauer, Max Kevane-Campbell, Martin Kuhns, Sönke Andres, Stefan Niemann, Thomas A. Wichelhaus, Nils Wetzstein

**Affiliations:** 1 Molecular and Experimental Mycobacteriology, Research Center Borstel, Leibniz Lung Center 28413, Borstel, Germany; 2 National and WHO Supranational Reference Laboratory for Mycobacteria, Research Center Borstel, Leibniz Lung Center 28413, Borstel, Germany; 3 Leibniz Research Alliance INFECTIONS, Borstel, Germany; 4 Department of Internal Medicine, Infectious Diseases, Goethe University Frankfurt, University Hospital 9173, Frankfurt am Main, Germany; 5 Mycobacterial Infection Research Unit (MIRU), Goethe University Frankfurt 9173, Frankfurt am Main, Germany; 6 Department of Radiology, Goethe University Frankfurt, University Hospital 9173, Frankfurt am Main, Germany; 7 EPHE, PSL University 316338, Paris, France; 8 Institut de Systématique, Evolution, Biodiversité, ISYEB, Muséum national d’Histoire naturelle, CNRS, Sorbonne Université, EPHE, Université des Antilles 27063, Paris, France; 9 Institute of Medical Microbiology and Infection Control, Goethe University Frankfurt, University Hospital 9173, Frankfurt am Main, Germany; University of Western Australia, Perth, Australia

**Keywords:** NTM, non-tuberculous mycobacteria, *Mycobacterium xenopi*, slow-growing mycobacteria, antimicrobial resistance

## Abstract

*Mycobacterium xenopi* causes non-tuberculous mycobacterial pulmonary disease (NTM-PD) that is difficult to treat. However, data on the genomic population structure, antimicrobial susceptibility, and the clinical significance of this pathogen remain scarce. We analyzed 76 clinical *M. xenopi* isolates from 70 patients collected between 1995 and 2020 in Frankfurt am Main, Germany. All isolates underwent phenotypic drug susceptibility testing and whole-genome sequencing. Cluster analysis, including isolates from this study and all hitherto available high-quality *M. xenopi* genome data sets in the Sequence Read Archive (*n* = 11), was performed by core genome multilocus sequence typing. In our cohort, only 26.5% of patients met criteria for clinically relevant NTM-PD. Phylogenetic analysis identified three large hospital-associated clusters (≤10 allelic difference), each involving between 7 and 20 patients and persisting for over 18 years, suggesting prolonged transmission chains or a common environmental source. We also defined three major clades (≤50 allelic difference), two of which contained isolates from the United Kingdom. Clofazimine and guideline-recommended antimycobacterial agents showed good *in vitro* efficacy, except rifampicin, with 23.6% resistance. This study represents a major expansion of *M. xenopi* genomic resources and provides insights into the genomic population structure, phenotypic susceptibility, and clinical characteristics of *M. xenopi*. Guideline-recommended antimycobacterials show good *in vitro* activity, while clofazimine may be a valuable addition to *M. xenopi* therapy. The identified clusters underscore the need for further investigation into transmission dynamics and globally successful clones.

**IMPORTANCE:**

*Mycobacterium xenopi* is an increasingly recognized opportunistic lung pathogen that is difficult to treat. Infections often occur in patients with pre-existing health conditions and can present substantial diagnostic and therapeutic challenges. A deeper understanding of its genetic diversity and resistance mechanisms is essential for optimal patient management and for clarifying potential transmission routes. By analyzing 76 whole-genome sequences together with detailed clinical information and phenotypic drug-susceptibility data, this study substantially expands the available genomic repertoire for *M. xenopi*. While clinical relevance was limited in our cohort, most guideline-recommended antimicrobial agents showed good efficacy *in vitro*. The detection of closely related strains might point toward a common environmental source of infection. These findings highlight the need for continued surveillance and provide a comprehensive foundation that supports more accurate monitoring, improved understanding of disease behavior, and future investigations into *M. xenopi* pathogenicity.

## INTRODUCTION

*Mycobacterium xenopi* is a slow-growing non-tuberculous mycobacterium (NTM) (1). It has first been isolated from the African clawed frog (*Xenopus laevis*) in 1959 (2). *M. xenopi* can cause pulmonary disease (NTM-PD) in patients with predisposing lung conditions but also extrapulmonary disease (3, 4). In Europe, *M. xenopi* is a rare pathogen causing NTM-PD, but there are regional differences (5). In the South of Germany, it seems to be more frequently isolated than in the North (6). Still, the exact prevalence of *M. xenopi* disease and its global trends over time remain largely unknown, mainly due to the absence of mandatory reporting and the diagnostic challenges common to NTM infections.

To diagnose NTM-PD and distinguish it from contamination or colonization, the American Thoracic Society (ATS)/ERS/IDSA/ESCMID criteria are applied (1, 7). These criteria encompass microbiological (at least two positive sputum cultures or one positive bronchoalveolar lavage [BAL]), clinical (pulmonary symptoms and exclusion of alternative diagnoses), and radiological findings (such as cavities or nodules), all of which must be met to confirm disease. Applying these criteria, the clinical relevance of *M. xenopi* seems to vary between different geographical locations; for example, 27.9% of isolates in Croatia fulfill the diagnostic criteria, compared with 75.6% in France (8, 9). Still, recent ATS/ERS/IDSA/ESCMID guidelines classify *M. xenopi* as a pathogen associated with high mortality (1).

Like many other NTM, *M. xenopi* infection is supposed to take place via uptake from the environment. It has been shown to be present in indoor and outdoor water sources like hot water systems in hospitals and stream waters (10, 11) but has also been isolated from animals like birds (12). Human-to-human transmission of *M. xenopi* lung infection has not yet been demonstrated to date. In fact, a recent study investigating a potential outbreak of *M. xenopi* in 6 patients attending the same hospital found that the isolates were not clonal as assessed by using whole genome sequencing (WGS) (13). In comparison to other NTM, such as *M. abscessus* and *M. avium* complex (MAC), whole-genome data for *M. xenopi* are limited, with only 11 non-redundant, high-quality WGS data sets available at the time of writing. Consistent with this, there is currently no available information on the genomic population structure of *M. xenopi*.

Therapy of infections caused by *M. xenopi* is difficult, and optimal therapeutic regimens are not well defined due to low case numbers and lack of randomized controlled studies. The current ATS guidelines recommend a daily regimen consisting of at least three drugs: rifampicin (or rifabutin), ethambutol, and either a macrolide (azithromycin or clarithromycin) and/or fluoroquinolone (e.g., moxifloxacin). Parenteral amikacin can be added for severe disease. The optimal treatment duration is unknown, but guidelines suggest treatment to be continued for at least 12 months after culture conversion (1). Treatment outcomes have been unsatisfactory, with a recent meta-analysis showing only 32.0% (CI 16.5%–49.8 %) of culture conversion in subjects with NTM-PD caused by *M. xenopi* (14). Phenotypic drug susceptibility testing of *M. xenopi* remains a challenge due to its fastidious growth and a lack of suitable breakpoints for most antibiotic substances (15). Only a few studies have correlated *in vitro* drug susceptibility data with treatment outcomes (16, 17); therefore, no recommendation is given for or against susceptibility-based treatment of *M. xenopi* disease (1). On a genomic level, the resistance determinants or virulence genes of the pathogen are not well characterized, and in contrast to MAC or *M. abscessus*, there are no commercialized line probe assays for the detection of genotypic resistance against macrolides or aminoglycosides (18).

With this genomic study on *M. xenopi* isolates from a cohort of patients at a German tertiary care center, we aim to provide the first steps to unraveling the genomic population structure of the pathogen, to investigate its genotypic and phenotypic drug susceptibility, and to correlate these findings with the available clinical data.

## MATERIALS AND METHODS

### Included isolates from this study

All available *M. xenopi* isolates obtained from patients treated at the University Hospital Frankfurt between 1995 and 2020 were included. Initial species identification was performed as part of the clinical routine with 16S rRNA sequencing, ITS-PCR, or by line probe assay using the Genotype Mycobacterium CM (Hain Lifescience GmbH, Nehren, Germany) (19). For patients who were treated after 2006, clinical data were extracted using our local patient information system (ORBIS, Dedalus Healthcare, Bonn, Germany), including clinical manifestation, antimycobacterial therapy, fulfillment of the ATS/ERS/IDSA/ESCMID criteria, and comorbidities (such as HIV infection and other immunosuppression). If a high-resolution CT score of the lung was available, the images were assessed by a radiologist according to the CT score by Song et al. (20). This study was approved by our local ethics committee under file number 2022-672.

### Culture and WGS

Isolates were cultivated on Middlebrook 7H10 agar until visible growth was detected, and DNA was extracted as described previously using the CTAB method (21). Next-generation sequencing libraries were generated from extracted genomic DNA with a modified Illumina Nextera library kit protocol. Subsequently, libraries were sequenced in a 2× 150-bp paired-end run on an Illumina NextSeq 500 or 2000 instrument (Illumina, San Diego, CA, USA).

### Bioinformatic analysis

Raw reads from our isolates as well as from publicly available *M. xenopi* isolates downloaded on 1 August 2023 underwent quality control using fastqc and multiqc (22, 23). For public data with only genome assemblies available, raw reads were simulated with dwgsim v. 0.1.12-13 (24). The species was confirmed with NTM-Profiler v 0.2.2 (25). Contamination with DNA of other bacterial species was assessed using Kraken v 2.1.2 with the standard database (29.04.2021) (26). All reads with low sequencing quality, identified as another species than *M. xenopi* by NTMprofiler, or more than 5% reads of another bacterial species were excluded from further bioinformatic analysis (Fig. S1).

Average nucleotide identities (ANIs) were calculated for isolates in comparison to the reference strain *M. xenopi* JCM 15661, as well as for all isolate-to-isolate comparisons using fastANI (27). For phylogenetic analysis and cluster detection, an *ad hoc* core genome multilocus sequence typing (cgMLST) scheme for *M. xenopi* was created using the complete genome of type strain *M. xenopi* JCM 15661 (accession number NZ_AP022314.1, 4716 genes) as the seed genome and all 87 genomes included in this study as penetration query genomes (File S1) in SeqSphere+ (28). The resulting scheme consisted of 2,565 core loci (comprising 54% of the total genes of the seed genome). Details on the development and validation of the cgMLST scheme, as well as on the determination of the thresholds, that is, genetic relatedness cutoffs, used for cluster analysis (10 and 50 alleles), are provided in the Supplementary Methods. D10 and D50 clusters represent groups of isolates in which any isolate is connected to at least one other isolate of the cluster by ≤10 or ≤50 allelic differences, respectively, based on the 2,565 analyzed genes. Genome assemblies were constructed using shovill v 1.1.0, with SKESA v 2.4.0 as the assembly algorithm (29). The neighbor-joining tree of 87 *M*. *xenopi* isolates was calculated from pairwise allelic distances calculated with cgMLST in SeqSphere+. Mashtree was used to generate a phylogenetic tree of German isolates with sufficient clinical metadata (30). ITol and ggtree were used to visualize metadata on the trees (31,32). Resistance and virulence genes were detected using AMRfinder v 3.11.2 with a reference database downloaded at 2023-09-26.1, a minimum identity for a blast-based hit of 50%, and a minimum required coverage of the reference gene of 50% (33). *RpoB* and *arr* sequence extraction, analysis, and phylogenetic tree construction were performed using BioNumerics v7.6.3.

### Phenotypic drug susceptibility testing (pDST)

pDST for 15 antibiotic agents was performed using Sensititre SLOMYCOI and SLOMYCO2 plates (Thermo Fisher Scientific, Waltham, USA) according to the manufacturer’s recommendations and CLSI guidelines (15, 34). This included the following antibiotic agents: clarithromycin, rifampicin, rifabutin, ethambutol, isoniazid, trimethoprim/sulfamethoxazole, amikacin, streptomycin, linezolid, ciprofloxacin, moxifloxacin, ethionamide, clofazimine, doxycycline, and minocycline. As no breakpoints for *M. xenopi* are available, breakpoints for slow-growing mycobacteria were derived from CLSI manual M62 (15).

### Statistical analysis

All statistical analyses were performed in R v. 4.3.1 (“Beagle Scouts”) using packages of the *tidyverse* (35, 36). Categorical variables are depicted as frequencies with percentage; numeric variables are depicted as mean with standard deviation for normally distributed data and median with an interquartile range (IQR) or range for non-normally distributed data. Differences in categorical variables between groups were assessed using the Fisher exact test. For these statistical tests, a significance level of alpha = 0.05 was used. Graphs were drawn using the *ggplot2* package (37).

## RESULTS

### Included isolates and sequence data

Overall, 87 isolates including the reference strain *M. xenopi* JCM 15661 were included for genomic analysis (Fig. S1). The majority of genome sequences were generated in this study (*n* = 76), while 11 public samples were included (Table 1; Table S1). Public strains consisted of seven isolates from the UK, three from the USA, and one from the Netherlands. Except for the type strain (isolated from the frog *Xenopus laevis*), all isolates were of human origin (86/87, 98.9%). The majority of human samples with a known sample type were isolated from respiratory specimens (38/40, 95%), while two samples were extrapulmonary (2/40, 5%). For 46/87 samples (52.9%), the sample type was not available. From our hospital, 69 primary isolates and 7 sequential isolates were included.

**TABLE 1:** Isolates from which genomic sequences were included into the study

	n/N (%)
Species	
*M. xenopi*	87/87 (100.0)
Country of origin	
Germany	76/87 (87.4)
Netherlands	1/87 (1.1)
United Kingdom	7/87 (8.0)
United States of America	1/87 (1.1)
Isolation material	
Human samples	86/87 (98.9)
Pulmonary samples	38/87 (43.7)
BAL	11/87 (12.6)
Bronchial secretion (aspirated during bronchoscopy)	14/87 (16.1)
Sputum	12/87 (13.8)
Lung tissue	1/87 (1.1)
Extrapulmonary samples	
Vertebral biopsy	2/87 (2.3)
Clinical sample not specified	46/87 (52.9)
Zoonotic sample	1/87 (1.1)
Time point of isolation	
Primary	69/87 (79.3)
Sequential isolates	7/87 (8.0)
Not known	10/87 (11.5)
Category	
Frankfurt samples	76/87 (87.4)
Public samples	11/87 (12.6)

### Genome characteristics and phylogenetic relations

All genome sequences displayed more than 98.4% ANI with the reference genome *M. xenopi* JCM 15661. The minimum ANI between isolates in the whole data set was 98.77%. Mean GC content was 65.9% (range, 65.8%–66.2%).

**Fig 1:**
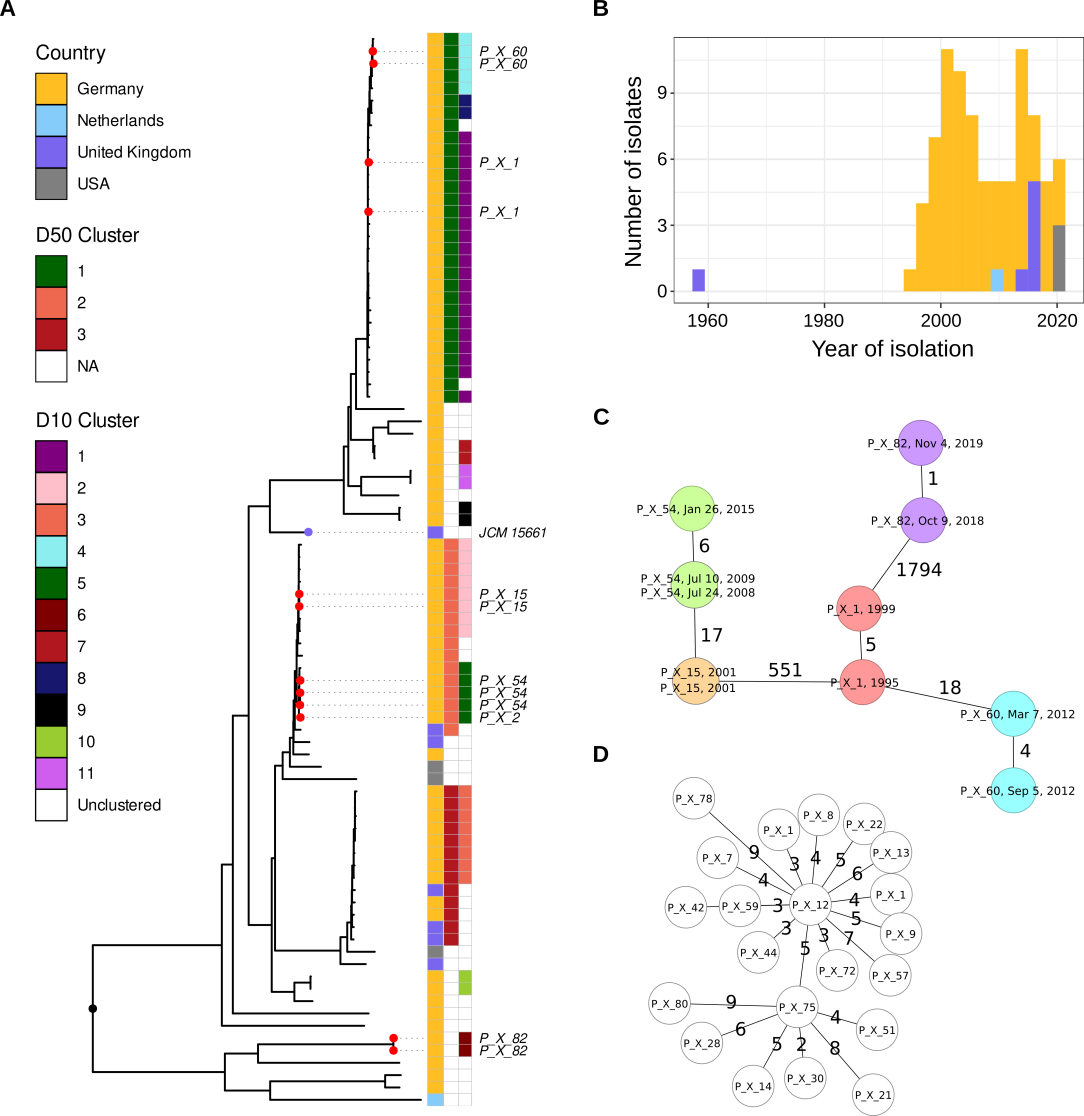
(**A**) Neighbor-joining tree based on cgMLST pairwise allele distances of 87 included *Mycobacterium xenopi* isolates (76 from this study and 11 public ones). Sequential isolates, collected from the same patient over time, and reference strain are denoted by red and purple dots, respectively. All non-German isolates in this study were retrieved from public repositories. Only the three largest D50 clusters are colored. (**B**) Timeline of isolates used in this study. (**C**) Minimum spanning tree of all patients for which sequential isolates were available. Each patient is represented by a distinct color. Labels indicate patient ID and the collection date of the isolate. (**D**) Minimum spanning tree of the largest D10 cluster comprising 21 isolates from Frankfurt. Allele distances between two isolates are displayed on the branches (not to scale).

Median cgMLST distance between isolates from different patients at our center was 556 alleles (IQR, 209–745; range, 0–1,878 alleles). The median allele distance between isolates from the same patient was 4, with a maximum distance of 6 alleles observed between isolates collected from the same patient 6 years apart (Fig. 1). For cluster analysis, a threshold of 10 alleles (D10) was applied to the full data set (87 isolates), identifying 11 clusters with 59 (67.8%) clustered isolates from 53 patients and 28 (32.2%) unclustered isolates from 28 patients (Fig. 1). A total of 10 out of 11 D10 clusters contained isolates from different patients, indicating potential human-to-human transmission or infection/contamination from the same source (Table S1; Fig. S2). All D10 clusters consisted of isolates from patients from this study only, and no public genomes grouped with the local isolates. The largest cluster (Cluster 1) comprised 21 strains from Frankfurt, isolated from 20 different patients over a 23-year period (1995–2018) (Fig. 1). Clusters 2 and 3 comprised isolates from 7 and 8 different patients, collected over periods of 19 and 18 years, respectively. The remaining clusters comprised isolates from 4 or fewer patients (Fig. 1; Table S1). The median allele distance between public and local genomes was 622 alleles (IQR, 333–713; range, 11–1,949).

Next to the D10 clusters, we could also observe three major clades in the cgMLST-based phylogeny comprising isolates that could be connected with less than 50 alleles (D50) (Fig. 1). Of those, one clade consisted only of local isolates, but two others contained isolates from the UK, as well. Further, not all isolates from the USA and UK grouped together in the phylogenetic tree, respectively. Besides the three major D50 clusters, five additional smaller D50 clusters (each containing a maximum of two isolates) were identified, while 18 isolates remained unclustered at this threshold (Table S1).

### Genotypic and phenotypic drug susceptibility

AMRfinder detected the presence of only three resistance genes in all 87 *M*. *xenopi* isolates: *aac(2')-Ic* (aminoglycoside N-acetyltransferase, associated with aminoglycoside resistance), *arr* (NAD(+)--rifampin ADP-ribosyltransferase Arr-2b, associated with rifampicin resistance), and *narA* (ionophore ABC transporter ATP-binding subunit, associated with maduramicin, narasin, and aslinomycin resistance).

**Fig 2:**
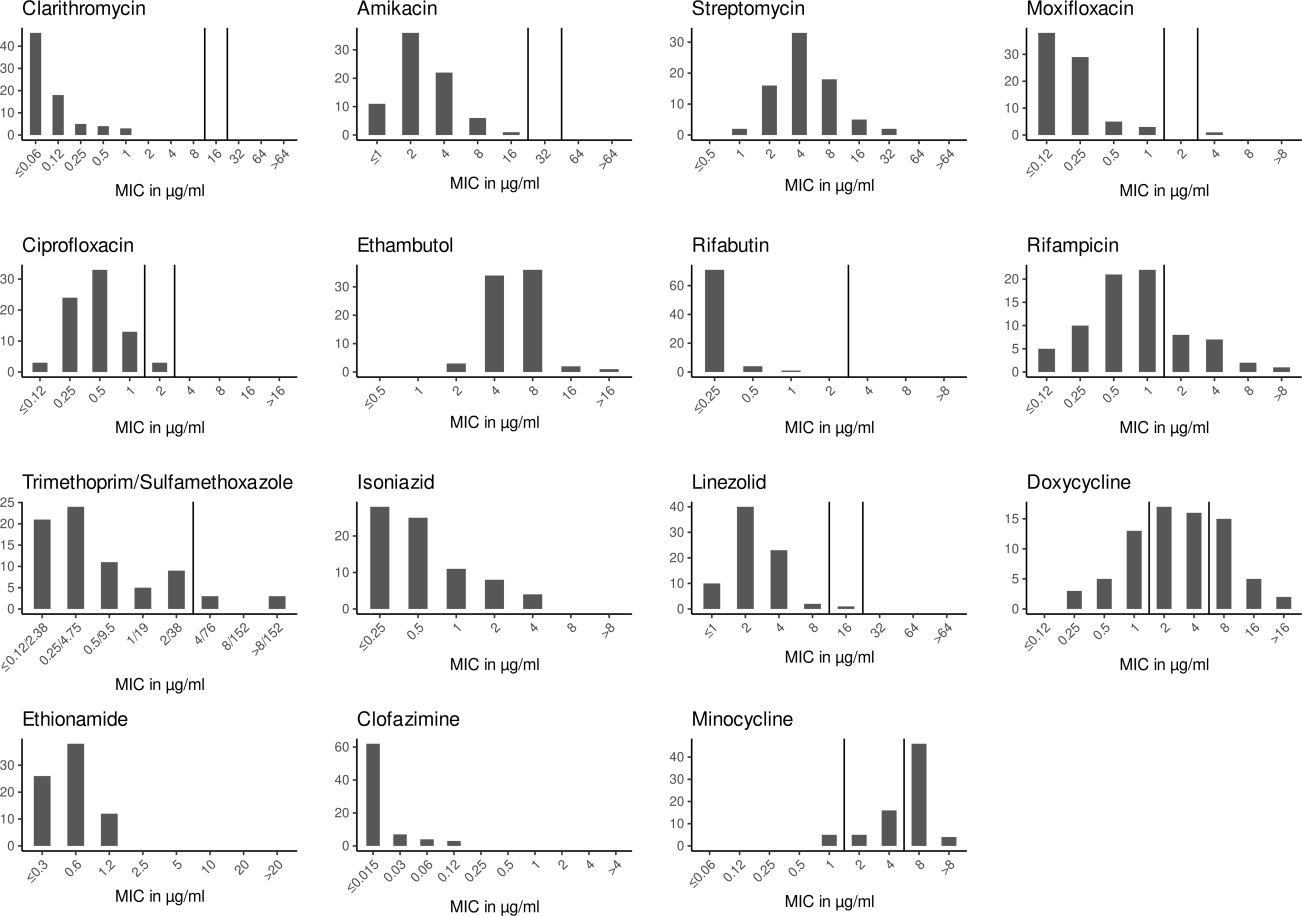
MIC distributions for included strains (*n* = 76) using SLOMYCO1 and SLOMYCO2 testing systems. Vertical lines depict breakpoints if available.

However, we could not detect any phenotypic macrolide or aminoglycoside resistances in our cohort of 76 *M*. *xenopi* isolates (MIC_90_ for clarithromycin 0.25 µg/mL and for amikacin 4 µg/mL, Table S2; Fig. 2). Further, rifabutin showed good *in vitro* efficacy (MIC_90_≤0.12 µg/mL), while 23.6% (18/76) of isolates were phenotypically resistant to rifampicin (MIC_90_= 4 µg/mL). Phylogenetic analysis of the *rpoB* gene (associated with rifampicin resistance) did not result in clustering of all strains with higher minimum inhibitory concentrations (MICs) for rifampicin (Fig. S3). Moreover, the rpoB amino acid sequence was identical across all *M. xenopi* isolates in this study, including the type strain, with the exception of a single public isolate that carried one amino acid substitution. The rifampin ADP-ribosyltransferase Arr-2b amino acid sequences of 4 out of 18 isolates resistant to rifampicin carried a H44N mutation (Fig. S4), whereas one susceptible isolate had a truncated Arr-2b due to a premature stop codon. However, sequential isolates obtained from the same patient showed no differences in their ADP-ribosyltransferase amino acid sequences, even when their susceptibility profiles differed (Fig. S5).

Isolates displayed considerably low MICs to linezolid, ethionamide, and fluoroquinolones. MICs for doxycycline and minocycline were high (MIC_90_= 8 µg/mL each). Finally, clofazimine had good efficacy against all *M. xenopi* isolates (MIC_90_= 0.03 µg/mL) *in vitro*. Further, we did not observe substantial differences in categorical susceptibility in patients with serial isolates (Fig. S5).

### Clinical characteristics of *M. xenopi* infection

The median age of the 36 included patients with sufficient available metadata was 52 years (IQR, 45.8–63.0). The majority were males (80.6%, *n* = 29) (Table 2). The most frequent comorbidities were an underlying structural lung disease (52.8%, *n* = 19) and/or HIV (58.3%, *n* = 21). Further, more than half of the patients were smokers (61.1%, *n* = 22) or suffered from alcohol abuse (12/36, 33.3%). Interestingly, not even a single isolate was recovered from a patient with cystic fibrosis (CF). Only a fraction of the patients received immunosuppressive therapies (mainly prednisone in 3/36 patients, 8.3%). At the end of the respective observation period, six patients were deceased (16.7%; median time to death 17 months [range, 0–93 months]).

**TABLE 2:** Baseline characteristics, comorbidities, and risk factors of patients with available data at University Hospital Frankfurt

	All	HIV	Non-HIV	*P*-value
	n/N (%)	n/N (%)	n/N (%)	(HIV vs Non-HIV)
Overall manifestations				
Pulmonary	34/36 (94.4)	19/21 (90.5)	15/15 (100.0)	0.5
Extrapulmonary	1/36 (2.8)	1/21 (4.8)	0/15 (0.0)	1
Disseminated	1/36 (2.8)	1/21 (4.8)	0/15 (0.0)	1
Comorbidities				
CF	0/36 (0.0)	0/21 (0.0)	0/15 (0.0)	1
Malignancy	8/36 (22.2)	5/21 (23.8)	3/15 (20.0)	1
Structural lung disease	19/36 (52.8)	8/21 (38.1)	11/15 (73.3)	0.05
Rheumatic disease	0/36 (0.0)	0/21 (0.0)	0/15 (0.0)	1
Others	27/36 (75.0)	14/21 (66.7)	13/15 (86.7)	0.25
Smoker	22/36 (61.1)	16/21 (76.2)	6/15 (40.0)	0.04
Alcohol abuse	12/36 (33.3)	10/21 (47.6)	2/15 (13.3)	0.04
Isolation of other mycobacterial species				
*M. tuberculosis*	1/36 (2.8)	0/21 (0.0)	1/15 (6.7)	0.42
*M. kansasii*	1/36 (2.8)	1/21 (4.8)	0/15 (0.0)	1
*M. avium*	1/36 (2.8)	1/21 (4.8)	0/15 (0.0)	0.42
ATS criteria				
ATS criteria positive	9/34 (26.5)	5/19 (26.3)	4/15 (26.7)	1
Pulmonary symptoms and appropriate radiology	25/34 (73.5)	15/19 (78.9)	10/15 (66.6)	0.46
Exclusion of other diagnosis	9/34 (26.5)	5/19 (26.3)	4/15 (26.7)	1
Two positive sputa	4/34 (11.8)	2/19 (10.5)	2/15 (13.3)	1
Positive BAL	24/34 (70.6)	14/19 (73.7)	10/15 (66.7)	0.72
Positive bronchial biopsy	3/34 (8.8)	1/19 (5.3)	2/15 (13.3)	0.57
Received NTM effective therapy	13/36 (36.1)	10/21 (47.6)	3/15 (20.0)	0.16
Outcome				
Deceased	6/36 (16.7)	4/21 (19.0)	2/15 (13.3)	1

**Fig 3:**
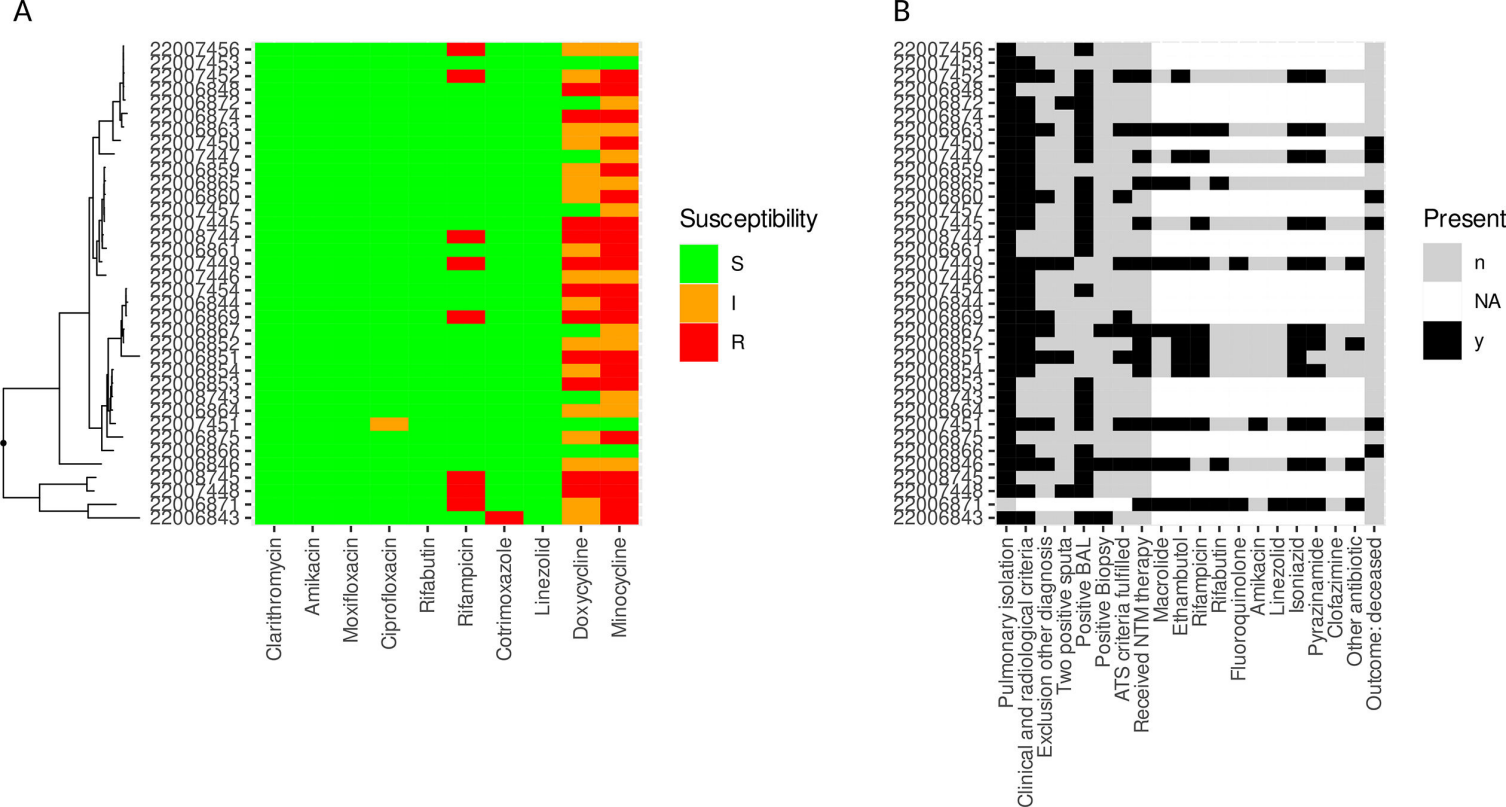
(**A**) Phenotypic drug susceptibility results obtained using Sensititre SLOMYCO1 and SLOMYCO2 plates for 36 patients with sufficient clinical metadata. (**B**) Fulfillment of criteria for NTM disease according to the international ATS guidelines and treatment data for these 36 patients. The phylogenetic tree in panel A was generated with mashtree.

The majority of patients had pulmonary infection or colonization with *M. xenopi* (94.4%, *n* = 34), 1 patient had isolated extrapulmonary infection (2.8%), and 1 patient suffered from disseminated disease with isolation from more than 2 body sites (2.8%) (Fig. 3). In patients with pulmonary isolation of *M. xenopi,* ATS criteria were fulfilled in 9/34 patients (26.5%). However, 25 patients displayed pulmonary symptoms and appropriate radiology. In 24 patients, *M. xenopi* was isolated from BAL. Fulfillment of ATS criteria was similar in non-HIV patients and HIV patients (26.7% vs 26.3%). Isolates from patients fulfilling ATS criteria did not cluster together in a phylogenetic tree (Fig. 3). In patients with available CT scans of the thorax (*n* = 26), the radiological score described by Song et al. was higher in patients without an HIV infection than in those with HIV (median 9.5 vs median 3.5, *P* = 0.05, Fig. S6). The two extrapulmonary manifestations were described in patients with an HIV infection. Patients without HIV were more likely to suffer from a pre-existing structural lung disease (73.3% vs 38.1%, *P* = 0.049).

A total of 13 patients received antimycobacterial therapy, including the 2 patients with extra-pulmonary affection and disseminated infection. The median treatment duration was 19 months (ranging from 6 to 30 months). Therapy consisted mainly of macrolides, ethambutol, and a rifamycin (Fig. 3). When evaluated by phenotypic susceptibility (Fig. 3A), only 2 patients received an inefficient drug based on CLSI breakpoints (2 times rifampicin, Fig. 3B). Culture conversion was observed in 9 patients, while for the rest, no adequate follow-up cultures were available. A total of 2 patients of those treated died during the observation period. Death was not associated with therapeutic mismatches or antibiotic resistance.

## DISCUSSION

*M. xenopi* is a rare cause of NTM-PD and extrapulmonary infections. Up to date, clinico-genomic data on this pathogen are scarce. In this study, we provide a first overview of the genomic population structure of the pathogen. Hospital-associated clusters point toward the potential of local human-to-human transmission, a common environmental source, or environmental contamination. The combination with phenotypic drug susceptibility and clinical data might help future investigations of this opportunistic pathogen causing NTM-PD.

Overall, we show a rather good susceptibility to common antimycobacterial drugs in *M. xenopi*. Nearly all therapeutic agents recommended by guidelines could be considered efficacious *in vitro* (1). This was especially the case for azithromycin, fluoroquinolones, rifabutin, and amikacin. However, rifampicin showed higher MICs, as observed in many other NTMs (38). In MAC, this has led to questioning its role in the treatment of MAC pulmonary disease (39). On the other hand, clofazimine could be considered a useful addition to a treatment regimen based on the excellent in *vitro* efficacy in our cohort and is also currently adopted into many other NTM treatment regimens at the moment (40). Nevertheless, the low MICs should also be interpreted with regards to the ideal incubation temperature of *M. xenopi* (some isolates grow better at 42°C–45°C) (41) and the incubation temperature of the commercial Sensititre drug susceptibility testing plate (37°C). Therefore, our MIC distributions might underestimate antibiotic resistance. Further, rifampicin has been shown to be unstable in liquid media used by the current pDST methods for slow-growing mycobacteria (42), and breakpoints provided by CLSI are currently under scrutiny. This has to be taken into account when interpreting these results.

In *M. tuberculosis*, rifampicin resistance is almost exclusively associated with amino acid substitutions in the β-subunit of RNA polymerase (RpoB), particularly within the well-characterized rifampicin resistance–determining region. Such mutations directly alter the binding site of rifampicin and reliably explain phenotypic resistance (43). In contrast, among the *M. xenopi* isolates analyzed here, the RpoB amino acid sequence was highly conserved, and no mutations were observed that could account for elevated MIC values. However, we identified a H44N mutation in a rifampin ADP-ribosyltransferase (Arr-2b) in 4 out of 18 resistant isolates and none of the susceptible isolates. This protein has also been reported to confer intrinsic rifampicin resistance in *M. smegmatis* (44) and *M. abscessus* (45). However, to the best of our knowledge, mutations in this gene have not previously been reported or analyzed, and further investigation is warranted to determine whether this represents a novel resistance mechanism for rifampicin in *M. xenopi*.

To date, only a single complete *M. xenopi* genome is available in NCBI (type strain JCM 15661), consisting of one circular 4.9 Mbp chromosome without plasmids. Our study expands the currently limited genomic data set for *M. xenopi*, adding 76 short-read data sets to the 11 previously available good-quality public short-read data sets and assemblies (draft and complete genomes).

Due to the absence of sufficient public genomes, a global population structure could not be established. Nonetheless, our cgMLST-based phylogeny revealed 3 large D50 clades, at least 2 of which include isolates from multiple countries similar to patterns reported for *M. abscessus* (46, 47). The presence of several larger D10 clusters (up to 21 closely related isolates) with strains collected over extended time periods (up to 23 years) may indicate prolonged local human-to-human transmission or, more likely, a shared environmental source, such as local drinking water supplies, or a common source of contamination (e.g., contaminated bronchoscopes and tap water within the hospital or reagentia) (48), though we could not confirm this in the absence of detailed epidemiological data**⁠**. However, in 2022, 11 water samples were collected from various locations in our hospital (offices, bathrooms, laboratories, etc.d ), but no mycobacteria were isolated from these samples (49). These findings suggest that either no environmental source within the hospital explains the observed hospital-associated *M. xenopi* clusters identified in our study, or that the specific source was not captured by the sampling performed. Additional environmental sampling and surveillance would be valuable to investigate this further.

In our cohort, clinically relevant pulmonary disease defined by the ATS criteria was present in 26.5% of patients with available metadata. This lies in the range of previous studies (albeit lower than in previous European studies) and underlines the geographic variation of NTM pathogenicity (4, 8, 9). These differences might be caused by different bacterial populations and differential host susceptibilities to disease, but also by different clinical practices in disease diagnosis and management. Whether *M. xenopi* has a lower virulence than other NTM species warrants further investigation. Interestingly, the most common predisposition in our cohort was an HIV infection, and previous studies at our center have shown that *M. xenopi* is the most common NTM after MAC isolated from respiratory specimens in this cohort (50). Although we did observe relatively similar rates of fulfilled diagnostic criteria in patients with HIV and those without, interestingly, radiological changes were objectively less pronounced in HIV patients, which might be linked to a less severe inflammatory response to the pathogen.

This study has several limitations: first, we only had limited clinical data available as the first isolates were included from 1995, but clinical records were available from 2005 onward. Second, being a monocentric study, we can only give limited information on the global extent of clusters and the global population structure of *M. xenopi*. This should be part of future studies. Third, as the data set did only contain a few phenotypic resistant strains to antimycobacterial agents recommended by guidelines, we could not screen for known or novel resistance gene mutations in *M. xenopi,* except for rifampicin.

### Conclusion

This study provides an insight into the genomic population structure, phenotypic susceptibility, and clinical characteristics of *M. xenopi* isolates from Frankfurt, Germany. We observe local clustering and a good overall efficacy of antimycobacterial agents *in vitro,* except for rifampicin, while clofazimine might be a good addition to currently recommended regimens. In our cohort, only one quarter of patients fulfilled criteria for clinically relevant pulmonary disease. The global population structure of this pathogen and its association with clinical phenotypes will have to be investigated in future studies.

## Data Availability

The sequence data generated in this project can be accessed at the European Nucleotide Archive (ENA) under ENA project number PRJEB95836. Accession numbers for all samples are available in Table S1.

## SUPPLEMENTAL MATERIAL

File S1 jcm.01511-25-s0001.txt Creation of cgMLST scheme.

Supplemental figures jcm.01511-25-s0002.docx Figures S1 to S6.

Supplemental methods jcm.01511-25-s0003.docx Supplemental methods.

Table S1 jcm.01511-25-s0004.xlsx Included *M. xenopi* genomes and metadata for phylogenetic analysis.

Table S2 jcm.01511-25-s0005.xlsx Phenotypic drug susceptibility data: MIC50, MIC90, and susceptibility category (if available) for 76 *M. xenopi* isolates from University hospital Frankfurt.

ASM does not own the copyrights to Supplemental Material that may be linked to, or accessed through, an article. The authors have granted ASM a non-exclusive, world-wide license to publish the Supplemental Material files. Please contact the corresponding author directly for reuse.

## References

[1] Daley CL, Iaccarino JM, Lange C, Cambau E, Wallace RJ Jr, Andrejak C, Böttger EC, Brozek J, Griffith DE, Guglielmetti L, Huitt GA, Knight SL, Leitman P, Marras TK, Olivier KN, Santin M, Stout JE, Tortoli E, van Ingen J, Wagner D, Winthrop KL. 2020. Treatment of nontuberculous mycobacterial pulmonary disease: an official ATS/ERS/ESCMID/IDSA clinical practice guideline. Eur Respir J 56:2000535. doi:10.1183/13993003.00535-2020 32636299 PMC8375621

[2] Schwabacher H. 1959. A strain of Mycobacterium isolated from skin lesions of a cold-blooded animal, Xenopus laevis, and its relation to atypical acid-fast bacilli occurring in man. J Hyg (Lond) 57:57–67. doi:10.1017/s0022172400019896 13641655 PMC2218100

[3] Manfredi R, Nanetti A, Tadolini M, Calza L, Morelli S, Ferri M, Marinacci G. 2003. Role of Mycobacterium xenopi disease in patients with HIV infection at the time of highly active antiretroviral therapy (HAART). Comparison with the pre-HAART period. Tuberculosis (Edinb) 83:319–328. doi:10.1016/s1472-9792(03)00053-2 12972345

[4] van Ingen J, Boeree MJ, de Lange WCM, Hoefsloot W, Bendien SA, Magis-Escurra C, Dekhuijzen R, van Soolingen D. 2008. Mycobacterium xenopi clinical relevance and determinants, the Netherlands. Emerg Infect Dis 14:385–389. doi:10.3201/eid1403.061393 18325251 PMC2570832

[5] Dahl VN, Laursen LL, He Y, Zhang YA, Wang MS. 2023. Species distribution among patients with nontuberculous mycobacteria pulmonary disease in Europe. J Infect 87:469–472. doi:10.1016/j.jinf.2023.03.010 36913984

[6] Corbett C, Finger P, Heiß-Neumann M, Bohnert J, Eder IB, Eisele M, Friesen I, Kaasch AJ, Kehrmann J, Lang R, Rödel J, Roessler S, Schmidt A, Schneitler S, Schui D, Schuler F, Sedlacek L, Serr A, Sitaru A-G, Steinmann J, Wagner D, Wichelhaus TA, Hofmann-Thiel S, Hoffmann H, EpiNTM-Group. 2023. Development of prevalence and incidence of non-tuberculous mycobacteria in German laboratories from 2016 to 2020. Emerg Microbes Infect 12:2276342. doi:10.1080/22221751.2023.2276342 37883336 PMC10769520

[7] Griffith DE, Aksamit T, Brown-Elliott BA, Catanzaro A, Daley C, Gordin F, Holland SM, Horsburgh R, Huitt G, Iademarco MF, Iseman M, Olivier K, Ruoss S, von Reyn CF, Wallace RJ, Winthrop K. 2007. An official ATS/IDSA statement: diagnosis, treatment, and prevention of nontuberculous mycobacterial diseases. Am J Respir Crit Care Med 175:367–416. doi:10.1164/rccm.200604-571ST 17277290

[8] Jankovic M, Sabol I, Zmak L, Jankovic VK, Jakopovic M, Obrovac M, Ticac B, Bulat LK, Grle SP, Marekovic I, Samarzija M, van Ingen J. 2016. Microbiological criteria in non-Tuberculous mycobacteria pulmonary disease: a tool for diagnosis and epidemiology. Int J Tuberc Lung Dis 20:934–940. doi:10.5588/ijtld.15.0633 27287647

[9] Andréjak C, Lescure F-X, Pukenyte E, Douadi Y, Yazdanpanah Y, Laurans G, Schmit J-L, Jounieaux V. 2009. Mycobacterium xenopi pulmonary infections: a multicentric retrospective study of 136 cases in North-East France. Thorax 64:291–296. doi:10.1136/thx.2008.096842 19052044

[10] Sebakova H, Kozisek F, Mudra R, Kaustova J, Fiedorova M, Hanslikova D, Nachtmannova H, Kubina J, Vraspir P, Sasek J. 2008. Incidence of nontuberculous mycobacteria in four hot water systems using various types of disinfection. Can J Microbiol 54:891–898. doi:10.1139/w08-080 18997845

[11] Torkko P, Suomalainen S, Iivanainen E, Suutari M, Tortoli E, Paulin L, Katila ML. 2000. Mycobacterium xenopi and related organisms isolated from stream waters in Finland and description of Mycobacterium botniense sp. nov. Int J Syst Evol Microbiol 50:283–289. doi:10.1099/00207713-50-1-283 10826815

[12] Ledwoń A, Napiórkowska A, Augustynowicz-kopeć E, Szeleszczuk P. 2018. Drug susceptibility of non-tuberculous strains of Mycobacterium isolated from birds from Poland. Pol J Microbiol 67:487–492. doi:10.21307/pjm-2018-057 30550235 PMC7256868

[13] Kling K, Osborn R, Menon A, Williams J, Cardew R, Al-Heeti O, Santoiemma P, Angarone M, Gatesy S, Kochan T, Zembower T, Krueger K, Ozer EA, Qi C. 2023. A cluster of six respiratory cultures positive for Mycobacterium xenopi -Clinical characteristics and genomic characterization. J Clin Tuberc Other Mycobact Dis 33:100397. doi:10.1016/j.jctube.2023.100397 37727871 PMC10505978

[14] Diel R, Ringshausen FC, Richter E, Welker L, Schmitz J, Nienhaus A. 2017. Microbiological and clinical outcomes of treating non-Mycobacterium avium complex nontuberculous mycobacterial pulmonary disease: a systematic review and meta-analysis. Chest 152:120–142. doi:10.1016/j.chest.2017.04.166 28461147

[15] Woods GL, Wengenack NL, Grace Lin D, Barbara Brown-Elliott MA, Daniela Maria Cirillo M, Conville PS, et al.. 2018. M62: performance standards for susceptibility testing of mycobacteria, Nocardia spp., and other aerobic actinomycetes. https://clsi.org/31339680

[16] Andréjak C, Lescure F-X, Douadi Y, Laurans G, Smail A, Duhaut P, Jounieaux V, Schmit J-L. 2007. Non-tuberculous mycobacteria pulmonary infection: management and follow-up of 31 infected patients. J Infect 55:34–40. doi:10.1016/j.jinf.2007.01.008 17360040

[17] Smith MJ, Citron KM. 1983. Clinical review of pulmonary disease caused by Mycobacterium xenopi. Thorax 38:373–377. doi:10.1136/thx.38.5.373 6879487 PMC459561

[18] GenoType NTM-DR. 2018. Detection of NTM resistances. https://www.bruker.com/en/products-and-solutions/molecular-diagnostics/assays/mycobacteria/genotype-ntm-dr.html

[19] GenoType Mycobacterium CM. 2023. Detection and differentiation of NTM. https://www.hain-lifescience.de/en/products/microbiology/mycobacteria/ntm/genotype-mycobacterium-cm.html

[20] Song JW, Koh W-J, Lee KS, Lee JY, Chung MJ, Kim TS, Kwon OJ. 2008. High-resolution CT findings of Mycobacterium avium-intracellulare complex pulmonary disease: correlation with pulmonary function test results. Am J Roentgenol 191:W160–W166. doi:10.2214/AJR.07.3505 18806143

[21] De Almeida IN, Da Silva Carvalho W, Rossetti ML, Costa ERD, De Miranda SS. 2013. Evaluation of six different DNA extraction methods for detection of Mycobacterium tuberculosis by means of PCR-IS6110: preliminary study. BMC Res Notes 6:561. doi:10.1186/1756-0500-6-561 24373461 PMC3891981

[22] GitHub-s-andrews. 2023. FastQC: a quality control analysis tool for high throughput sequencing data. https://github.com/s-andrews/FastQC

[23] Ewels P, Magnusson M, Lundin S, Käller M. 2016. MultiQC: summarize analysis results for multiple tools and samples in a single report. Bioinformatics 32:3047–3048. doi:10.1093/bioinformatics/btw354 27312411 PMC5039924

[24] GitHub - nh13. 2023. DWGSIM: whole genome simulator for next-generation sequencing. https://github.com/nh13/DWGSIM

[25] GitHub - jodyphelan. 2023. NTM-Profiler: profiling NTM WGS data. https://github.com/jodyphelan/NTM-Profiler

[26] Wood DE, Lu J, Langmead B. 2019. Improved metagenomic analysis with Kraken 2. Genome Biol 20:257. doi:10.1186/s13059-019-1891-0 31779668 PMC6883579

[27] GitHub - ParBLiSS. 2023. FastANI: fast whole-genome similarity (ANI) estimation. https://github.com/ParBLiSS/FastANI

[28] Jünemann S, Sedlazeck FJ, Prior K, Albersmeier A, John U, Kalinowski J, Mellmann A, Goesmann A, von Haeseler A, Stoye J, Harmsen D. 2013. Updating benchtop sequencing performance comparison. Nat Biotechnol 31:294–296. doi:10.1038/nbt.2522 23563421

[29] GitHub - tseemann. 2023. shovill: assemble bacterial isolate genomes from Illumina paired-end reads. https://github.com/tseemann/shovill

[30] Katz LS, Griswold T, Morrison SS, Caravas JA, Zhang S, den Bakker HC, Deng X, Carleton HA. 2019. Mashtree: a rapid comparison of whole genome sequence files. J Open Source Softw 4:1762. doi:10.21105/joss.01762 PMC9380445 35978566

[31] Letunic I, Bork P. 2021. Interactive Tree Of Life (iTOL) v5: an online tool for phylogenetic tree display and annotation. Nucleic Acids Res 49:W293–W296. doi:10.1093/nar/gkab301 33885785 PMC8265157

[32] Yu G, Smith D, Zhu H, Guan Y, Lam T-Y. 2017. ggtree: an R package for visualization and annotation of phylogenetic trees with their covariates and other associated data. Methods Ecol Evol 8:28–36. doi:10.1111/2041-210X.12628

[33] Feldgarden M, Brover V, Gonzalez-Escalona N, Frye JG, Haendiges J, Haft DH, Hoffmann M, Pettengill JB, Prasad AB, Tillman GE, Tyson GH, Klimke W. 2021. AMRFinderPlus and the Reference Gene Catalog facilitate examination of the genomic links among antimicrobial resistance, stress response, and virulence. Sci Rep 11:12728. doi:10.1038/s41598-021-91456-0 34135355 PMC8208984

[34] Clinical Laboratory Standards Institute. 2018. Susceptibility testing of Mycobacteria, Nocardia Spp., and other aerobic Actinomycetes 3rd Ed. Wayne, PA Clinical Laboratory Standards Institute 31339680

[35] R Core Team. 2018. R: a language and environment for statistical computing. https://www.r-project.org/.

[36] Wickham H, Averick M, Bryan J, Chang W, McGowan L, François R, Grolemund G, Hayes A, Henry L, Hester J, Kuhn M, Pedersen T, Miller E, Bache S, Müller K, Ooms J, Robinson D, Seidel D, Spinu V, Takahashi K, Vaughan D, Wilke C, Woo K, Yutani H. 2019. Welcome to the tidyverse. J Open Source Softw 4:1686. doi:10.21105/joss.01686

[37] Wickham H. 2009. Ggplot2: elegant graphics for data analysis. Springer-Verlag New York.

[38] Wetzstein N, Kohl TA, Andres S, Schultze TG, Geil A, Kim E, Biciusca T, Hügel C, Hogardt M, Lehn A, Vehreschild MJGT, Wolf T, Niemann S, Maurer FP, Wichelhaus TA. 2020. Comparative analysis of phenotypic and genotypic antibiotic susceptibility patterns in Mycobacterium avium complex. Int J Infect Dis 93:320–328. doi:10.1016/j.ijid.2020.02.059 32147539

[39] van Ingen J, Hoefsloot W, Dartois V, Dick T. 2024. Rifampicin has no role in treatment of Mycobacterium avium complex pulmonary disease and bactericidal sterilising drugs are needed: a viewpoint. Eur Respir J 63:2302210. doi:10.1183/13993003.02210-2023 38697635 PMC11063616

[40] Salillas S, Raaijmakers J, Aarnoutse RE, Svensson EM, Asouit K, van den Hombergh E, Te Brake L, Stemkens R, Wertheim HFL, Hoefsloot W, van Ingen J. 2024. Clofazimine as a substitute for rifampicin improves efficacy of Mycobacterium avium pulmonary disease treatment in the hollow-fiber model. Antimicrob Agents Chemother 68:e0115723. doi:10.1128/aac.01157-23 38259101 PMC10916390

[41] Wolinsky E. 1997. Editorial response: Is Mycobacterium xenopi an emerging pathogen? Clin Infect Dis 24:233–234. doi:10.1093/clinids/24.2.233 9114153

[42] Wetzstein N, Wichelhaus TA. 2022. Phenotypic drug susceptibility testing of rapid growing mycobacteria and the issue of antibiotic stability. Diagn Microbiol Infect Dis 102:115555. doi:10.1016/j.diagmicrobio.2021.115555 34678713

[43] Andre E, Goeminne L, Cabibbe A, Beckert P, Kabamba Mukadi B, Mathys V, Gagneux S, Niemann S, Van Ingen J, Cambau E. 2017. Consensus numbering system for the rifampicin resistance-associated rpoB gene mutations in pathogenic mycobacteria. Clin Microbiol Infect 23:167–172. doi:10.1016/j.cmi.2016.09.006 27664776

[44] Quan S, Venter H, Dabbs ER. 1997. Ribosylative inactivation of rifampin by Mycobacterium smegmatis is a principal contributor to its low susceptibility to this antibiotic. Antimicrob Agents Chemother 41:2456–2460. doi:10.1128/AAC.41.11.2456 9371349 PMC164144

[45] Rominski A, Roditscheff A, Selchow P, Böttger EC, Sander P. 2017. Intrinsic rifamycin resistance of Mycobacterium abscessus is mediated by ADP-ribosyltransferase MAB_0591. J Antimicrob Chemother 72:376–384. doi:10.1093/jac/dkw466 27999011

[46] Ruis C, Bryant JM, Bell SC, Thomson R, Davidson RM, Hasan NA, van Ingen J, Strong M, Floto RA, Parkhill J. 2021. Dissemination of Mycobacterium abscessus via global transmission networks. Nat Microbiol 6:1279–1288. doi:10.1038/s41564-021-00963-3 34545208 PMC8478660

[47] Diricks M, Merker M, Wetzstein N, Kohl TA, Niemann S, Maurer FP. 2022. Delineating Mycobacterium abscessus population structure and transmission employing high-resolution core genome multilocus sequence typing. Nat Commun 13:4936. doi:10.1038/s41467-022-32122-5 35999208 PMC9399081

[48] Bennett SN, Peterson DE, Johnson DR, Hall WN, Robinson-Dunn B, Dietrich S. 1994. Bronchoscopy-associated Mycobacterium xenopi pseudoinfections. Am J Respir Crit Care Med 150:245–250. doi:10.1164/ajrccm.150.1.8025757 8025757

[49] Diricks M, Frank D, Friesen I, Niemann S, Wichelhaus TA, Wetzstein N, NTMscope-Eco study group. 2025. Global lineages of non-tuberculous mycobacteria in residential water samples from Germany. BMC Microbiol 25:792. doi:10.1186/s12866-025-04563-7 41353231 PMC12701587

[50] Wetzstein N, Hügel C, Wichelhaus TA, Hogardt M, Eickmeier O, Küpper-Tetzel C-P, Kann G, Just-Nübling G, Stephan C, Wolf T. 2019. Species distribution and clinical features of infection and colonisation with non-tuberculous mycobacteria in a tertiary care centre, central Germany, 2006-2016. Infection 47:817–825. doi:10.1007/s15010-019-01317-2 31093923

