# Supplementary material for: Genomic population structure, antimicrobial susceptibility, and clinical features of Mycobacterium xenopi isolates, Frankfurt, Germany, 1995–2020
Source: J Clin Microbiol. 2026 Feb 9;64(3):e01511-25. doi: 10.1128/jcm.01511-25 (PMC12977513; doi:10.1128/jcm.01511-25)
Supplement: Supplemental figures — Figures S1 to S6. [file jcm.01511-25-s0002.docx]

**Supplementary figures:**


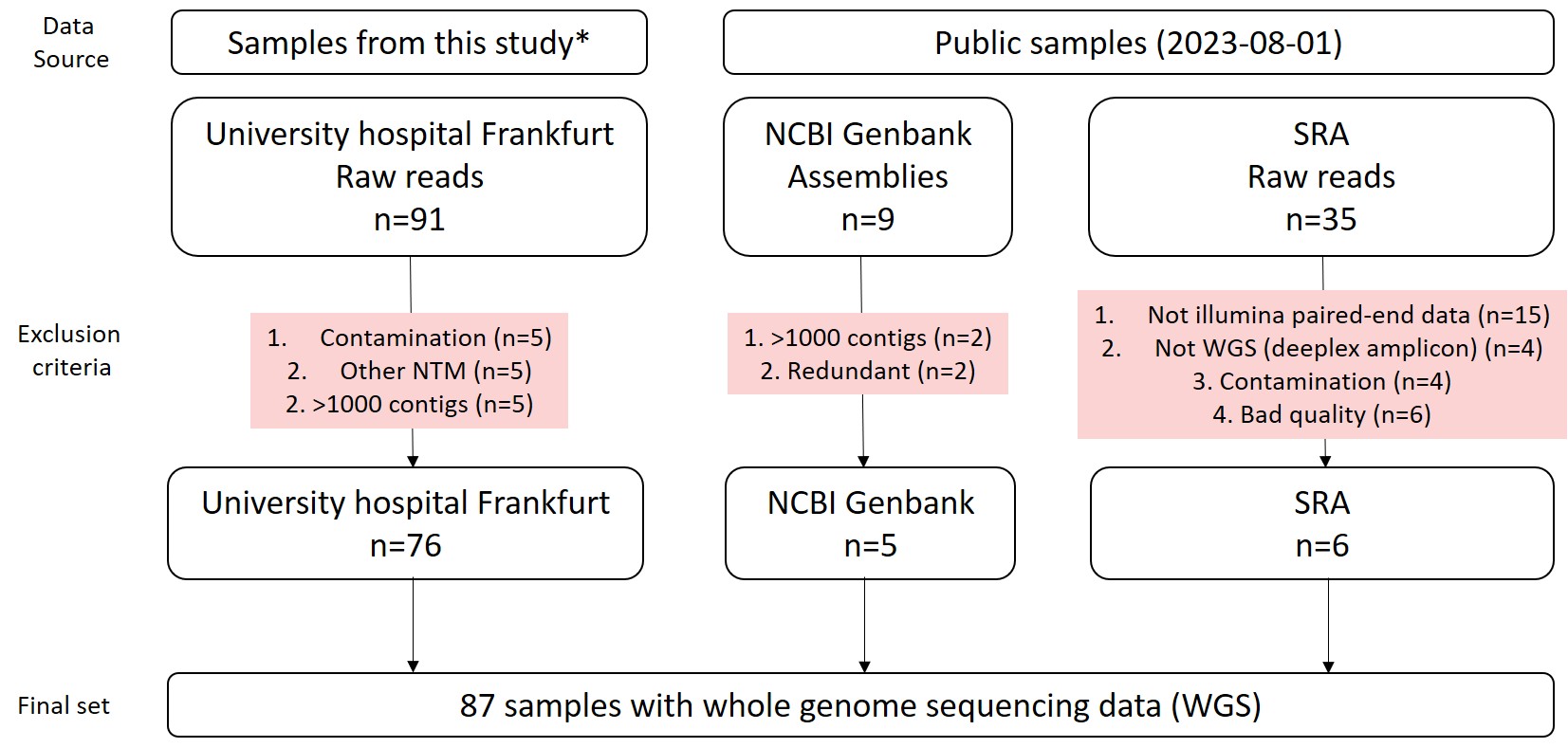
Figure S1: Workflow of isolate inclusion.


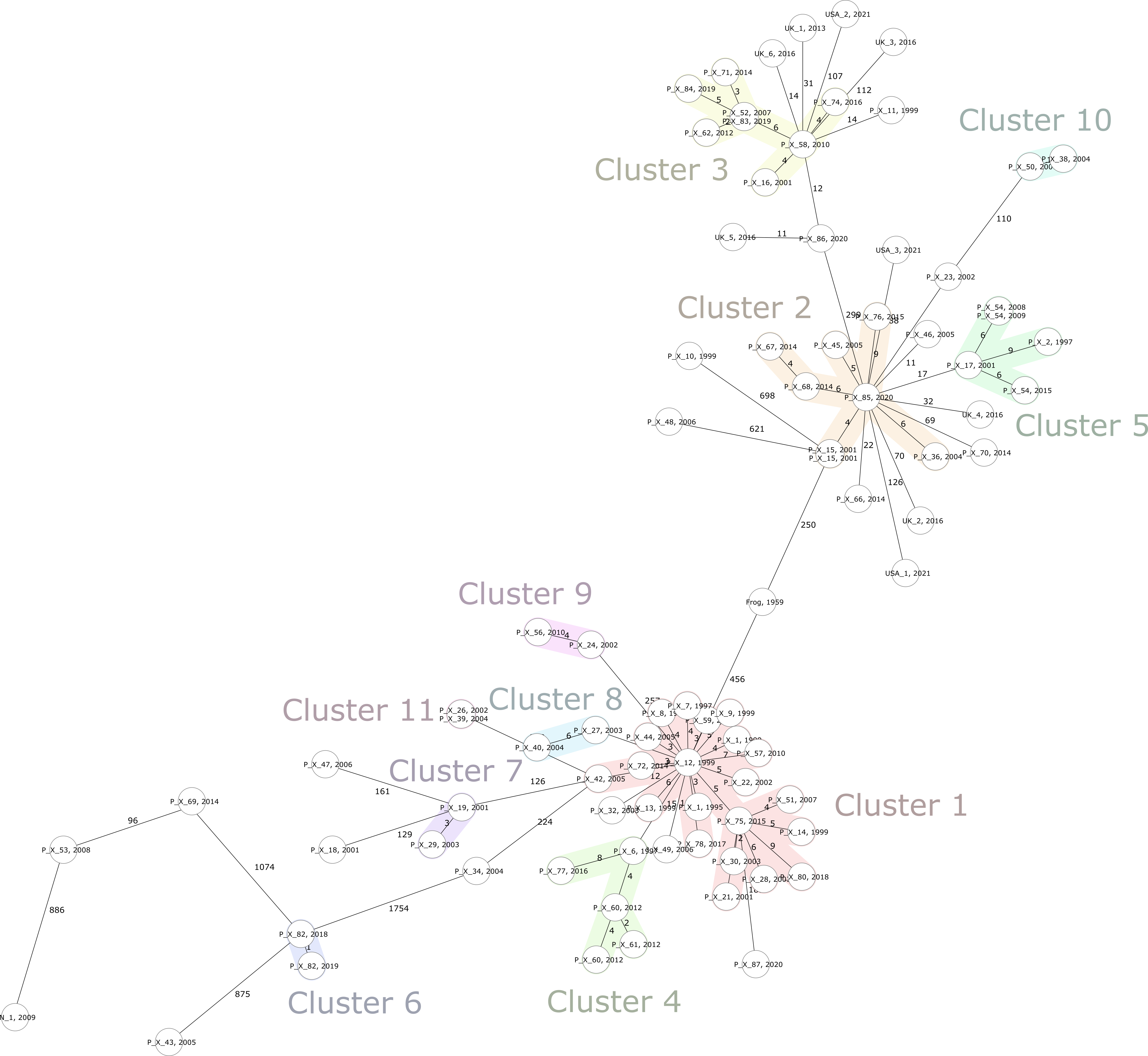


Figure S2: Minimum spanning tree of all included *M. xenopi* isolates (n=87) based on 2565 core loci. MST cluster distance threshold: 10 alleles. Figure generated in SeqSphere+.


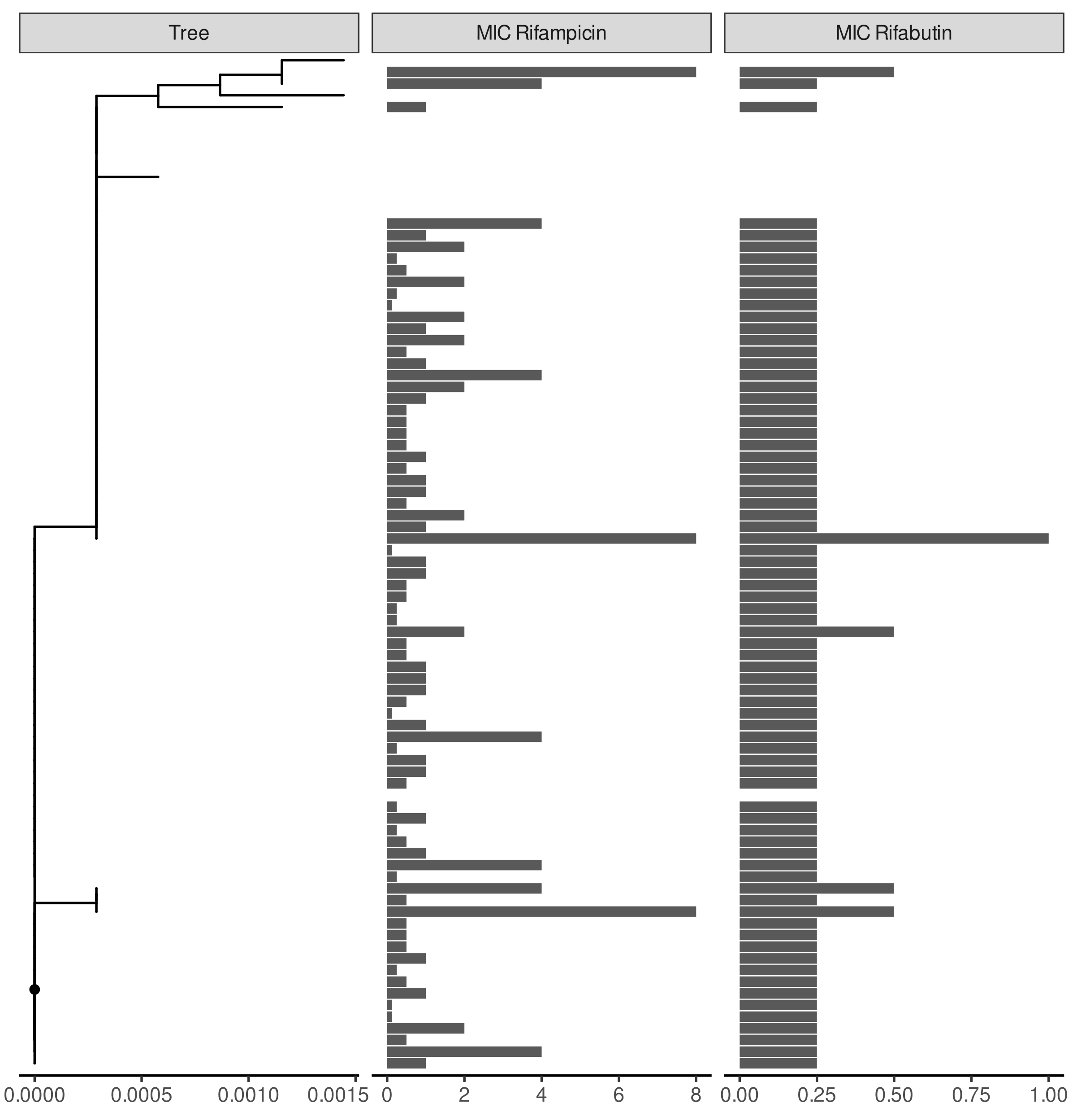


Figure S3: Phylogenetic tree of the *rpoB* gene and rifampicin and rifabutin minimum inhibitory concentrations (MIC) of 87 *M. xenopi* isolates. Strains are resistant for rifampicin and rifabutin if MIC ≥ 2 and MIC ≥ 4, respectively (according to CLSI guidelines M62 1st ed.). MICs were only available for isolates generated as part of this study,


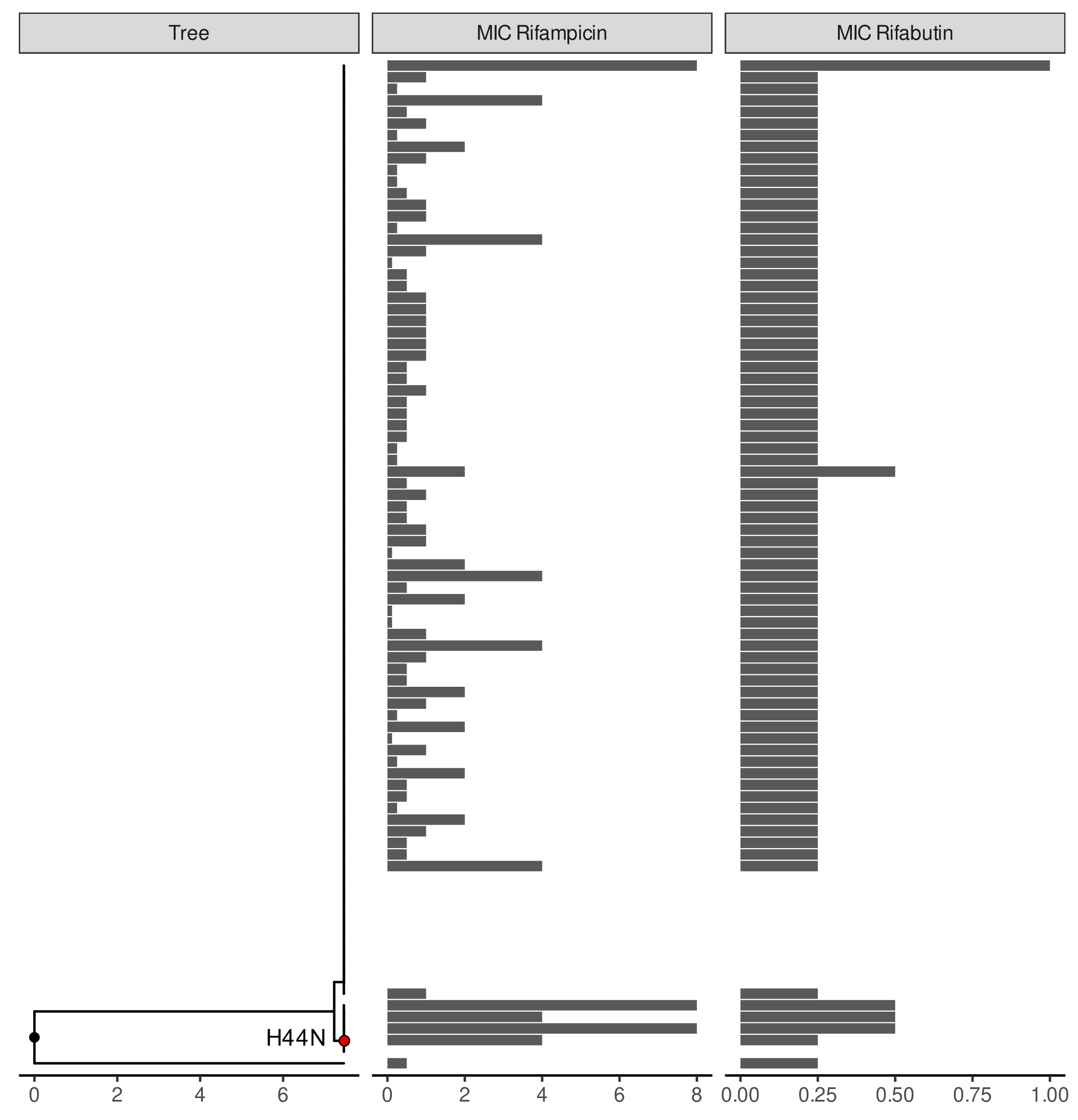


Figure S4: Phylogenetic tree of the ADP-ribosyltransferase Arr-2b amino acid sequence and rifampicin and rifabutin minimum inhibitory concentrations (MIC) for 87 *M. xenopi* isolates. Strains are resistant for rifampicin and rifabutin if MIC ≥ 2 and MIC ≥ 4, respectively (according to CLSI guidelines M62 1st ed.). MICs were only available for isolates generated as part of this study,


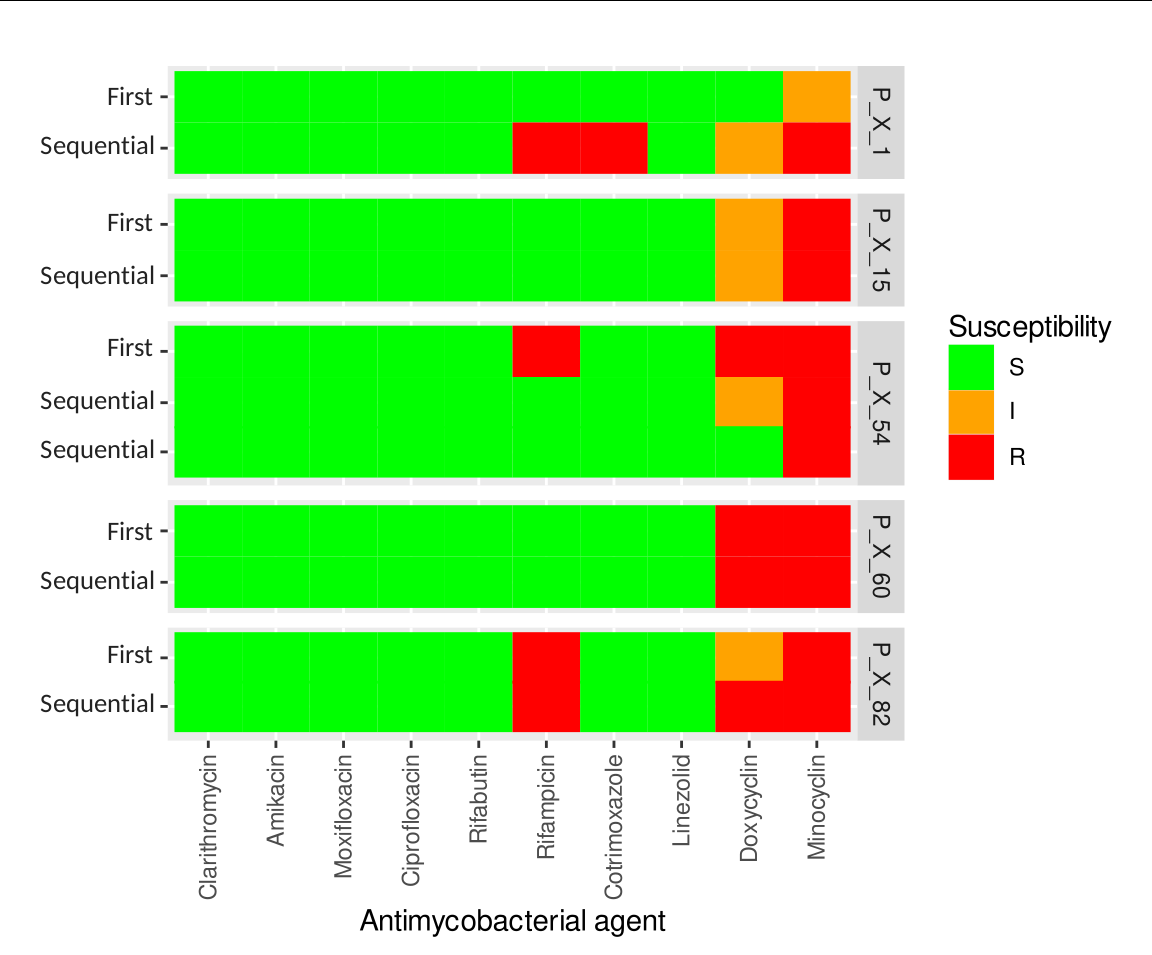
Figure S5: Phenotypic drug susceptibility results obtained using Sensititre^TM^ SLOMYCO and SLOMYCO2 plates for serial isolates of five patients.


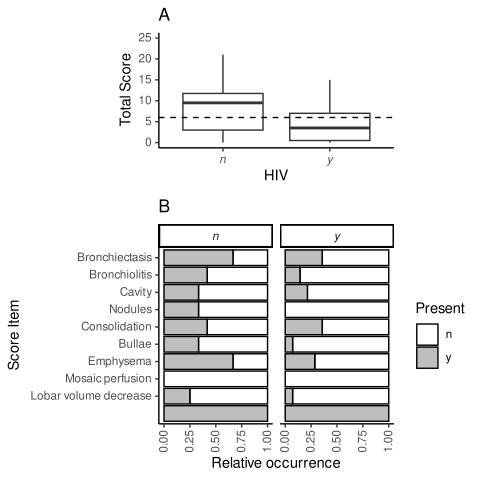
Figure S6: Radiological score by Song et al. differentiated by HIV status.
