# Supplementary material for: Genomic population structure, antimicrobial susceptibility, and clinical features of Mycobacterium xenopi isolates, Frankfurt, Germany, 1995–2020
Source: J Clin Microbiol. 2026 Feb 9;64(3):e01511-25. doi: 10.1128/jcm.01511-25 (PMC12977513; doi:10.1128/jcm.01511-25)
Supplement: Supplemental methods [file jcm.01511-25-s0003.docx]

Supplementary Methods for

**Genomic population structure, antimicrobial susceptibility and clinical features of *Mycobacterium xenopi* isolates, Frankfurt, Germany, 1995-2020**

Margo Diricks^1,2,3^, Lisa Marschall^4,5^, Teodora Biciusca^6^, Ann-Sophie Zielbauer^4^, Max Kevane-Campbell^4,5^, Martin Kuhns^2^, Sönke Andres^2^, Stefan Niemann^1,2,7,8^, Thomas A. Wichelhaus^5,9^, Nils Wetzstein^1,4,5^

Contents

[1 cgMLST scheme creation and validation 1](#_Toc214818005)

[1.1 Summary of resulting cgMLST scheme 1](#_Toc214818006)

[1.2 Selection of seed and penetration genomes 2](#_Toc214818007)

[1.3 Settings used to create and use the new cgMLST scheme 2](#_Toc214818008)

[1.3.1 Settings to create the penetration genomes 2](#_Toc214818009)

[1.3.2 Settings for scheme creation in SeqSphere+ 2](#_Toc214818010)

[1.3.3 Detailed progress of the cgMLST scheme creation process 3](#_Toc214818011)

[1.3.4 Settings for allele calling in SeqSphere+ 23](#_Toc214818012)

[1.4 Selection of threshold for recent transmission 24](#_Toc214818013)

[1.4.1 Analysis of type strain genomes 24](#_Toc214818014)

[1.4.2 Analysis of sequential isolates from the same patient 25](#_Toc214818015)

[1.5 Selection of threshold for potential dominant circulating clones 27](#_Toc214818016)

[1.6 Effect of assembler and choice of seed genome on cgMLST results 28](#_Toc214818017)

[2 References 31](#_Toc214818018)

# cgMLST scheme creation and validation

## Summary of resulting cgMLST scheme

**Seed genome:** Mycobacterium xenopi type strain JCM 15661 (NZ_AP022314.1)
**Penetration genomes:** 87 *M. xenopi* genomes draft or complete genomes (Supplementary Table S1).

**Core genes**: 2565 targets were defined for cgMLST (2299650 bases) = **cgMLST scheme**

**Accessory genes**: 1582 targets were identified as Accessory targets (1504119 bases)

**Discarded genes**: 569 targets were discarded

Please note that, for simplicity, the terms ‘loci,’ ‘targets,’ and ‘genes’ are used interchangeably in this text, although they are not strictly synonymous in a genomic context. The cgMLST scheme includes only coding sequences with defined start and stop codons; therefore, non-coding elements such as rRNA genes are not part of the scheme. For each target, each unique DNA sequence corresponds to one specific allele number, meaning that identical sequences share the same allele number, and any sequence variant receives a new number. All core genes, together with their allele sequences and assigned allele numbers, constitute the **cgMLST scheme**.

## Selection of seed and penetration genomes

The only complete genome available for the Mycobacterium xenopi type strain JCM 15661 (also known as ATCC 19250, NCTC 10042, or TMC 1482; accession number NZ_AP022314.1) was selected as the seed genome. This genome, which contains no plasmids, was assembled using Canu v1.8 with ONT MinION long-read sequencing data complemented by Illumina MiSeq short-read data^1^. Completeness according to checkM was 91%. As penetration genomes, all publicly available *M. xenopi* high-quality assemblies (n=5) and short read (i.e. Illumina) whole genome datasets (n=6) were used as well as 76 short read whole genome datasets generated as part of this study (Figure S1). Penetration genomes represent the collection of genomes used to define the core genome, i.e., the set of genes present in all or in the vast majority of strains of a species.

## Settings used to create and use the new cgMLST scheme

### Settings to create the penetration genomes

For the five publicly available NCBI assemblies, raw reads were simulated with dwgsim v. 0.1.12–13. For the other six public datasets, reads were downloaded from SRA using fasterq-dump. Then, all 87 *M. xenopi* read sets (including reads generated as part of this study) were assembled with shovill v 1.1.0 using SKESA v 2.4.0 as the assembly algorithm.

### Settings for scheme creation in SeqSphere+

The seed genome was downloaded using the function „Retrieve sequence from NCBI Genbank“ implemented in the target definer in SeqSphere+.

Seed Genome Gene Filters:

* Minimum Length Filter (gene requires >=50 bases)

* Start Codon Filter (requires start codon at beginning of the gene)

* Stop Codon Filter (requires single stop codon at end of gene)

* Homologous Gene Filter (requires no multiple copies of gene with BLAST overlap>=100bp, identity>=90.0%)

* Gene Overlap Filter (requires no overlap with other genes >4 bases)

Query Genome BLAST Search:

* Requires BLAST hit with overlap=100%, identity>=90.0% in every query genome

* BLAST options: Word size=11, Mismatch penalty=-1, Match reward=1, Gap open costs=5, Gap extension costs=2

Penetration Query Genomes Filters:

* Stop Codon Percentage Filter (requires single stop codon at end of gene in >80% penetration query genomes)

BLAST version 2.2.12

Citation: Altschul SF, Gish W, Miller W, Myers EW, Lipman DJ. 1990. Basic local alignment search tool. J Mol Biol 215:403-410.

### Detailed progress of the cgMLST scheme creation process

* cgMLST target search start at Jul 22, 2025, 11:18 AM

* Step 0: 4716 targets found in seed genome NZ_AP022314.1 (13-FEB-2025), 4917655 bases, 4716 genes (Mycobacterium xenopi strain JCM 15661 chromosome, complete genome)

* 3918 targets after filtering seed genome NZ_AP022314.1 (13-FEB-2025), 4917655 bases, 4716 genes (Mycobacterium xenopi strain JCM 15661 chromosome, complete genome)

* 3747 targets after blasting against Fasta file 22006822.fasta, 4775794 bases, 303 contigs

* 3695 targets after blasting against Fasta file 22006823.fasta, 4829318 bases, 223 contigs

* 3686 targets after blasting against Fasta file 22006824.fasta, 4739143 bases, 241 contigs

* 3676 targets after blasting against Fasta file 22006825.fasta, 4780900 bases, 256 contigs

* 3665 targets after blasting against Fasta file 22006826.fasta, 4735119 bases, 254 contigs

* 3659 targets after blasting against Fasta file 22006827.fasta, 4744373 bases, 180 contigs

* 3655 targets after blasting against Fasta file 22006828.fasta, 4895385 bases, 158 contigs

* 3652 targets after blasting against Fasta file 22006829.fasta, 4739944 bases, 220 contigs

* 3626 targets after blasting against Fasta file 22006830.fasta, 4829903 bases, 205 contigs

* 3567 targets after blasting against Fasta file 22006832.fasta, 4636660 bases, 237 contigs

* 3560 targets after blasting against Fasta file 22006833.fasta, 4771058 bases, 264 contigs

* 3466 targets after blasting against Fasta file 22006834.fasta, 4737943 bases, 428 contigs

* 3464 targets after blasting against Fasta file 22006835.fasta, 4719603 bases, 208 contigs

* 3454 targets after blasting against Fasta file 22006838.fasta, 4787283 bases, 240 contigs

* 3454 targets after blasting against Fasta file 22006839.fasta, 4641746 bases, 192 contigs

* 3447 targets after blasting against Fasta file 22006840.fasta, 4765093 bases, 295 contigs

* 3442 targets after blasting against Fasta file 22006842.fasta, 4734880 bases, 261 contigs

* 3062 targets after blasting against Fasta file 22006843.fasta, 4509132 bases, 215 contigs

* 3062 targets after blasting against Fasta file 22006844.fasta, 4779625 bases, 262 contigs

* 3061 targets after blasting against Fasta file 22006845.fasta, 4693677 bases, 204 contigs

* 2940 targets after blasting against Fasta file 22006846.fasta, 4650705 bases, 239 contigs

* 2932 targets after blasting against Fasta file 22006847.fasta, 4890714 bases, 226 contigs

* 2928 targets after blasting against Fasta file 22006848.fasta, 4770850 bases, 198 contigs

* 2918 targets after blasting against Fasta file 22006850.fasta, 4863670 bases, 279 contigs

* 2914 targets after blasting against Fasta file 22006851.fasta, 4716373 bases, 230 contigs

* 2911 targets after blasting against Fasta file 22006852.fasta, 4741863 bases, 203 contigs

* 2910 targets after blasting against Fasta file 22006853.fasta, 4865613 bases, 230 contigs

* 2902 targets after blasting against Fasta file 22006854.fasta, 4859791 bases, 255 contigs

* 2901 targets after blasting against Fasta file 22006855.fasta, 4858508 bases, 289 contigs

* 2900 targets after blasting against Fasta file 22006859.fasta, 4841097 bases, 216 contigs

* 2900 targets after blasting against Fasta file 22006860.fasta, 4841002 bases, 223 contigs

* 2894 targets after blasting against Fasta file 22006861.fasta, 4817753 bases, 286 contigs

* 2891 targets after blasting against Fasta file 22006863.fasta, 4723123 bases, 219 contigs

* 2890 targets after blasting against Fasta file 22006864.fasta, 4882178 bases, 235 contigs

* 2888 targets after blasting against Fasta file 22006865.fasta, 4835756 bases, 245 contigs

* 2877 targets after blasting against Fasta file 22006866.fasta, 4954652 bases, 272 contigs

* 2875 targets after blasting against Fasta file 22006867.fasta, 4741830 bases, 219 contigs

* 2870 targets after blasting against Fasta file 22006869.fasta, 4782648 bases, 228 contigs

* 2848 targets after blasting against Fasta file 22006871.fasta, 4455807 bases, 205 contigs

* 2844 targets after blasting against Fasta file 22006872.fasta, 4768496 bases, 229 contigs

* 2843 targets after blasting against Fasta file 22006873.fasta, 4459077 bases, 179 contigs

* 2836 targets after blasting against Fasta file 22006874.fasta, 4685913 bases, 244 contigs

* 2832 targets after blasting against Fasta file 22006875.fasta, 4908185 bases, 298 contigs

* 2832 targets after blasting against Fasta file 22007432.fasta, 4739431 bases, 182 contigs

* 2831 targets after blasting against Fasta file 22007433.fasta, 4787161 bases, 192 contigs

* 2809 targets after blasting against Fasta file 22007434.fasta, 4616618 bases, 156 contigs

* 2808 targets after blasting against Fasta file 22007435.fasta, 4740019 bases, 193 contigs

* 2806 targets after blasting against Fasta file 22007436.fasta, 4876670 bases, 165 contigs

* 2805 targets after blasting against Fasta file 22007437.fasta, 4714187 bases, 163 contigs

* 2805 targets after blasting against Fasta file 22007438.fasta, 4790701 bases, 185 contigs

* 2791 targets after blasting against Fasta file 22007439.fasta, 4776162 bases, 214 contigs

* 2791 targets after blasting against Fasta file 22007440.fasta, 4782281 bases, 187 contigs

* 2790 targets after blasting against Fasta file 22007441.fasta, 4743987 bases, 178 contigs

* 2769 targets after blasting against Fasta file 22007443.fasta, 4723802 bases, 209 contigs

* 2769 targets after blasting against Fasta file 22007444.fasta, 4848895 bases, 169 contigs

* 2768 targets after blasting against Fasta file 22007445.fasta, 4851032 bases, 152 contigs

* 2765 targets after blasting against Fasta file 22007446.fasta, 4873319 bases, 163 contigs

* 2765 targets after blasting against Fasta file 22007447.fasta, 4865777 bases, 192 contigs

* 2733 targets after blasting against Fasta file 22007448.fasta, 4259764 bases, 132 contigs

* 2732 targets after blasting against Fasta file 22007449.fasta, 4874133 bases, 162 contigs

* 2731 targets after blasting against Fasta file 22007450.fasta, 4852943 bases, 156 contigs

* 2729 targets after blasting against Fasta file 22007451.fasta, 4912731 bases, 220 contigs

* 2726 targets after blasting against Fasta file 22007452.fasta, 4770984 bases, 152 contigs

* 2726 targets after blasting against Fasta file 22007453.fasta, 4777448 bases, 129 contigs

* 2724 targets after blasting against Fasta file 22007454.fasta, 4784777 bases, 177 contigs

* 2721 targets after blasting against Fasta file 22007455.fasta, 4661800 bases, 143 contigs

* 2719 targets after blasting against Fasta file 22007456.fasta, 4779521 bases, 141 contigs

* 2717 targets after blasting against Fasta file 22007457.fasta, 4843279 bases, 164 contigs

* 2716 targets after blasting against Fasta file 22008738.fasta, 4785038 bases, 180 contigs

* 2716 targets after blasting against Fasta file 22008739.fasta, 4731282 bases, 132 contigs

* 2715 targets after blasting against Fasta file 22008740.fasta, 4729580 bases, 136 contigs

* 2714 targets after blasting against Fasta file 22008741.fasta, 4876104 bases, 175 contigs

* 2714 targets after blasting against Fasta file 22008742.fasta, 4717425 bases, 159 contigs

* 2707 targets after blasting against Fasta file 22008743.fasta, 4864410 bases, 210 contigs

* 2706 targets after blasting against Fasta file 22008744.fasta, 4831647 bases, 148 contigs

* 2704 targets after blasting against Fasta file 22008745.fasta, 4281485 bases, 135 contigs

* 2670 targets after blasting against Fasta file GCA0002577451.fasta, 4367395 bases, 74 contigs

* 2670 targets after blasting against Fasta file GCA0099362351-JCM15661.fasta, 4743306 bases, 146 contigs

* 2668 targets after blasting against Fasta file GCA0278541751.fasta, 4826004 bases, 122 contigs

* 2662 targets after blasting against Fasta file GCA0278541951.fasta, 4832413 bases, 129 contigs

* 2655 targets after blasting against Fasta file GCA0278542051.fasta, 4829124 bases, 150 contigs

* 2617 targets after blasting against Fasta file SRR1735675.fasta, 4785597 bases, 197 contigs

* 2605 targets after blasting against Fasta file SRR6045431.fasta, 4659968 bases, 197 contigs

* 2600 targets after blasting against Fasta file SRR6045630.fasta, 4703027 bases, 165 contigs

* 2592 targets after blasting against Fasta file SRR6046732.fasta, 4652636 bases, 202 contigs

* 2582 targets after blasting against Fasta file SRR6046822.fasta, 4911965 bases, 243 contigs

* 2579 targets after blasting against Fasta file SRR6046887.fasta, 4672594 bases, 205 contigs

* cgMLST target search ended at Jul 22, 2025, 11:42 AM

* 2565 targets after Penetration Query Genomes Stop Codon Percentage Filter

Figure SM1 Reduction in the number of remaining core targets (loci or genes) across the steps of scheme creation for Mycobacterium xenopi.

cgMLST Genome Coverage:

* 46.8% of Seed genome NZ_AP022314.1 (13-FEB-2025), 4917655 bases, 4716 genes with CDS (Mycobacterium xenopi strain JCM 15661 chromosome, complete genome) bases covered by cgMLST targets

* 48.2% of Query genome Fasta file 22006822.fasta bases covered by cgMLST targets

* 47.6% of Query genome Fasta file 22006823.fasta bases covered by cgMLST targets

* 48.5% of Query genome Fasta file 22006824.fasta bases covered by cgMLST targets

* 48.1% of Query genome Fasta file 22006825.fasta bases covered by cgMLST targets

* 48.6% of Query genome Fasta file 22006826.fasta bases covered by cgMLST targets

* 48.5% of Query genome Fasta file 22006827.fasta bases covered by cgMLST targets

* 47.0% of Query genome Fasta file 22006828.fasta bases covered by cgMLST targets

* 48.5% of Query genome Fasta file 22006829.fasta bases covered by cgMLST targets

* 47.6% of Query genome Fasta file 22006830.fasta bases covered by cgMLST targets

* 49.6% of Query genome Fasta file 22006832.fasta bases covered by cgMLST targets

* 48.2% of Query genome Fasta file 22006833.fasta bases covered by cgMLST targets

* 48.5% of Query genome Fasta file 22006834.fasta bases covered by cgMLST targets

* 48.7% of Query genome Fasta file 22006835.fasta bases covered by cgMLST targets

* 48.0% of Query genome Fasta file 22006838.fasta bases covered by cgMLST targets

* 49.5% of Query genome Fasta file 22006839.fasta bases covered by cgMLST targets

* 48.3% of Query genome Fasta file 22006840.fasta bases covered by cgMLST targets

* 48.6% of Query genome Fasta file 22006842.fasta bases covered by cgMLST targets

* 51.0% of Query genome Fasta file 22006843.fasta bases covered by cgMLST targets

* 48.1% of Query genome Fasta file 22006844.fasta bases covered by cgMLST targets

* 49.0% of Query genome Fasta file 22006845.fasta bases covered by cgMLST targets

* 49.4% of Query genome Fasta file 22006846.fasta bases covered by cgMLST targets

* 47.0% of Query genome Fasta file 22006847.fasta bases covered by cgMLST targets

* 48.2% of Query genome Fasta file 22006848.fasta bases covered by cgMLST targets

* 47.3% of Query genome Fasta file 22006850.fasta bases covered by cgMLST targets

* 48.8% of Query genome Fasta file 22006851.fasta bases covered by cgMLST targets

* 48.5% of Query genome Fasta file 22006852.fasta bases covered by cgMLST targets

* 47.3% of Query genome Fasta file 22006853.fasta bases covered by cgMLST targets

* 47.3% of Query genome Fasta file 22006854.fasta bases covered by cgMLST targets

* 47.3% of Query genome Fasta file 22006855.fasta bases covered by cgMLST targets

* 47.5% of Query genome Fasta file 22006859.fasta bases covered by cgMLST targets

* 47.5% of Query genome Fasta file 22006860.fasta bases covered by cgMLST targets

* 47.7% of Query genome Fasta file 22006861.fasta bases covered by cgMLST targets

* 48.7% of Query genome Fasta file 22006863.fasta bases covered by cgMLST targets

* 47.1% of Query genome Fasta file 22006864.fasta bases covered by cgMLST targets

* 47.6% of Query genome Fasta file 22006865.fasta bases covered by cgMLST targets

* 46.4% of Query genome Fasta file 22006866.fasta bases covered by cgMLST targets

* 48.5% of Query genome Fasta file 22006867.fasta bases covered by cgMLST targets

* 48.1% of Query genome Fasta file 22006869.fasta bases covered by cgMLST targets

* 51.6% of Query genome Fasta file 22006871.fasta bases covered by cgMLST targets

* 48.2% of Query genome Fasta file 22006872.fasta bases covered by cgMLST targets

* 51.6% of Query genome Fasta file 22006873.fasta bases covered by cgMLST targets

* 49.1% of Query genome Fasta file 22006874.fasta bases covered by cgMLST targets

* 46.9% of Query genome Fasta file 22006875.fasta bases covered by cgMLST targets

* 48.5% of Query genome Fasta file 22007432.fasta bases covered by cgMLST targets

* 48.0% of Query genome Fasta file 22007433.fasta bases covered by cgMLST targets

* 49.8% of Query genome Fasta file 22007434.fasta bases covered by cgMLST targets

* 48.5% of Query genome Fasta file 22007435.fasta bases covered by cgMLST targets

* 47.2% of Query genome Fasta file 22007436.fasta bases covered by cgMLST targets

* 48.8% of Query genome Fasta file 22007437.fasta bases covered by cgMLST targets

* 48.0% of Query genome Fasta file 22007438.fasta bases covered by cgMLST targets

* 48.1% of Query genome Fasta file 22007439.fasta bases covered by cgMLST targets

* 48.1% of Query genome Fasta file 22007440.fasta bases covered by cgMLST targets

* 48.5% of Query genome Fasta file 22007441.fasta bases covered by cgMLST targets

* 48.7% of Query genome Fasta file 22007443.fasta bases covered by cgMLST targets

* 47.4% of Query genome Fasta file 22007444.fasta bases covered by cgMLST targets

* 47.4% of Query genome Fasta file 22007445.fasta bases covered by cgMLST targets

* 47.2% of Query genome Fasta file 22007446.fasta bases covered by cgMLST targets

* 47.3% of Query genome Fasta file 22007447.fasta bases covered by cgMLST targets

* 54.0% of Query genome Fasta file 22007448.fasta bases covered by cgMLST targets

* 47.2% of Query genome Fasta file 22007449.fasta bases covered by cgMLST targets

* 47.4% of Query genome Fasta file 22007450.fasta bases covered by cgMLST targets

* 46.8% of Query genome Fasta file 22007451.fasta bases covered by cgMLST targets

* 48.2% of Query genome Fasta file 22007452.fasta bases covered by cgMLST targets

* 48.1% of Query genome Fasta file 22007453.fasta bases covered by cgMLST targets

* 48.1% of Query genome Fasta file 22007454.fasta bases covered by cgMLST targets

* 49.3% of Query genome Fasta file 22007455.fasta bases covered by cgMLST targets

* 48.1% of Query genome Fasta file 22007456.fasta bases covered by cgMLST targets

* 47.5% of Query genome Fasta file 22007457.fasta bases covered by cgMLST targets

* 48.1% of Query genome Fasta file 22008738.fasta bases covered by cgMLST targets

* 48.6% of Query genome Fasta file 22008739.fasta bases covered by cgMLST targets

* 48.6% of Query genome Fasta file 22008740.fasta bases covered by cgMLST targets

* 47.2% of Query genome Fasta file 22008741.fasta bases covered by cgMLST targets

* 48.7% of Query genome Fasta file 22008742.fasta bases covered by cgMLST targets

* 47.3% of Query genome Fasta file 22008743.fasta bases covered by cgMLST targets

* 47.6% of Query genome Fasta file 22008744.fasta bases covered by cgMLST targets

* 53.7% of Query genome Fasta file 22008745.fasta bases covered by cgMLST targets

* 52.7% of Query genome Fasta file GCA0002577451.fasta bases covered by cgMLST targets

* 48.5% of Query genome Fasta file GCA0099362351-JCM15661.fasta bases covered by cgMLST targets

* 47.7% of Query genome Fasta file GCA0278541751.fasta bases covered by cgMLST targets

* 47.6% of Query genome Fasta file GCA0278541951.fasta bases covered by cgMLST targets

* 47.6% of Query genome Fasta file GCA0278542051.fasta bases covered by cgMLST targets

* 48.1% of Query genome Fasta file SRR1735675.fasta bases covered by cgMLST targets

* 49.3% of Query genome Fasta file SRR6045431.fasta bases covered by cgMLST targets

* 48.9% of Query genome Fasta file SRR6045630.fasta bases covered by cgMLST targets

* 49.4% of Query genome Fasta file SRR6046732.fasta bases covered by cgMLST targets

* 46.8% of Query genome Fasta file SRR6046822.fasta bases covered by cgMLST targets

* 49.2% of Query genome Fasta file SRR6046887.fasta bases covered by cgMLST targets

**TARGET DETAILS**

**Seed Genome Start Codon Filter:** filtered out 66 targets (discarded)

MYXE_RS00680, MYXE_RS03155, MYXE_RS03185, MYXE_RS03705, MYXE_RS03820,

MYXE_RS04090, MYXE_RS04110, MYXE_RS04425, MYXE_RS04495, MYXE_RS04645,

MYXE_RS04835, MYXE_RS05050, MYXE_RS06860, MYXE_RS07135, MYXE_RS07195,

MYXE_RS07200, MYXE_RS07685, MYXE_RS08180, MYXE_RS09540, MYXE_RS10620,

MYXE_RS11325, MYXE_RS11400, MYXE_RS11510, MYXE_RS11640, MYXE_RS11920,

MYXE_RS12025, MYXE_RS14100, MYXE_RS14955, MYXE_RS15025, MYXE_RS16055,

MYXE_RS16925, MYXE_RS17350, MYXE_RS17980, MYXE_RS18095, MYXE_RS18270,

MYXE_RS18545, MYXE_RS19395, MYXE_RS19815, MYXE_RS20425, MYXE_RS20560,

MYXE_RS20970, MYXE_RS21040, MYXE_RS21760, MYXE_RS21765, MYXE_RS21915,

MYXE_RS22670, MYXE_RS22885, MYXE_RS23355, MYXE_RS23875, MYXE_RS24175,

MYXE_RS24305, MYXE_RS24360, MYXE_RS24415, MYXE_RS24480, MYXE_RS24580,

MYXE_RS24590, MYXE_RS24785, MYXE_RS24810, MYXE_RS24825, MYXE_RS25095,

MYXE_RS25160, MYXE_RS25170, MYXE_RS25175, MYXE_RS25225, MYXE_RS25330,

MYXE_RS25375

**Seed Genome Stop Codon Filter**: filtered out 386 targets (discarded)

MYXE_RS00065, MYXE_RS00130, MYXE_RS00275, MYXE_RS00320, MYXE_RS00390,

MYXE_RS00465, MYXE_RS00505, MYXE_RS00695, MYXE_RS00890, MYXE_RS00900,

MYXE_RS00995, MYXE_RS01000, MYXE_RS01060, MYXE_RS01175, MYXE_RS01220,

MYXE_RS01245, MYXE_RS01490, MYXE_RS01510, MYXE_RS01625, MYXE_RS01635,

MYXE_RS01670, MYXE_RS01690, MYXE_RS01735, MYXE_RS01745, MYXE_RS01800,

MYXE_RS01805, MYXE_RS01830, MYXE_RS01915, MYXE_RS01950, MYXE_RS01985,

MYXE_RS01995, MYXE_RS02065, MYXE_RS02180, MYXE_RS02200, MYXE_RS02355,

MYXE_RS02680, MYXE_RS02735, MYXE_RS02825, MYXE_RS02905, MYXE_RS03090,

MYXE_RS03100, MYXE_RS03160, MYXE_RS03210, MYXE_RS03245, MYXE_RS03260,

MYXE_RS03340, MYXE_RS03515, MYXE_RS03725, MYXE_RS03775, MYXE_RS03800,

MYXE_RS03805, MYXE_RS03815, MYXE_RS03865, MYXE_RS03975, MYXE_RS04005,

MYXE_RS04095, MYXE_RS04100, MYXE_RS04315, MYXE_RS04375, MYXE_RS04450,

MYXE_RS04505, MYXE_RS04510, MYXE_RS04665, MYXE_RS04770, MYXE_RS04775,

MYXE_RS04820, MYXE_RS04825, MYXE_RS04840, MYXE_RS04845, MYXE_RS04885,

MYXE_RS04900, MYXE_RS04975, MYXE_RS05075, MYXE_RS05305, MYXE_RS05485,

MYXE_RS05490, MYXE_RS05505, MYXE_RS05585, MYXE_RS05670, MYXE_RS05720,

MYXE_RS05975, MYXE_RS06070, MYXE_RS06080, MYXE_RS06090, MYXE_RS06225,

MYXE_RS06230, MYXE_RS06240, MYXE_RS06245, MYXE_RS06320, MYXE_RS06380,

MYXE_RS06510, MYXE_RS06585, MYXE_RS06780, MYXE_RS06820, MYXE_RS07015,

MYXE_RS07035, MYXE_RS07060, MYXE_RS07100, MYXE_RS07190, MYXE_RS07270,

MYXE_RS07285, MYXE_RS07405, MYXE_RS07415, MYXE_RS07425, MYXE_RS07625,

MYXE_RS07675, MYXE_RS07680, MYXE_RS07745, MYXE_RS07755, MYXE_RS07885,

MYXE_RS07895, MYXE_RS07925, MYXE_RS07975, MYXE_RS08205, MYXE_RS08260,

MYXE_RS08335, MYXE_RS08550, MYXE_RS08850, MYXE_RS08865, MYXE_RS09025,

MYXE_RS09045, MYXE_RS09175, MYXE_RS09230, MYXE_RS09445, MYXE_RS09560,

MYXE_RS09675, MYXE_RS09690, MYXE_RS09725, MYXE_RS09820, MYXE_RS10010,

MYXE_RS10030, MYXE_RS10035, MYXE_RS10065, MYXE_RS10135, MYXE_RS10170,

MYXE_RS10215, MYXE_RS10220, MYXE_RS10415, MYXE_RS10490, MYXE_RS10495,

MYXE_RS10560, MYXE_RS10665, MYXE_RS10710, MYXE_RS10820, MYXE_RS10870,

MYXE_RS11075, MYXE_RS11225, MYXE_RS11285, MYXE_RS11290, MYXE_RS11365,

MYXE_RS11405, MYXE_RS11410, MYXE_RS11555, MYXE_RS11570, MYXE_RS11635,

MYXE_RS11795, MYXE_RS11805, MYXE_RS11885, MYXE_RS11895, MYXE_RS12015,

MYXE_RS12020, MYXE_RS12040, MYXE_RS12080, MYXE_RS12220, MYXE_RS12255,

MYXE_RS12285, MYXE_RS12415, MYXE_RS12425, MYXE_RS12555, MYXE_RS12560,

MYXE_RS12600, MYXE_RS12610, MYXE_RS12645, MYXE_RS12650, MYXE_RS12695,

MYXE_RS12715, MYXE_RS12755, MYXE_RS12895, MYXE_RS12930, MYXE_RS12985,

MYXE_RS13135, MYXE_RS13330, MYXE_RS13365, MYXE_RS13390, MYXE_RS13435,

MYXE_RS13450, MYXE_RS13505, MYXE_RS13555, MYXE_RS13610, MYXE_RS13645,

MYXE_RS13800, MYXE_RS13850, MYXE_RS13875, MYXE_RS14055, MYXE_RS14070,

MYXE_RS14125, MYXE_RS14170, MYXE_RS14295, MYXE_RS14340, MYXE_RS14350,

MYXE_RS14390, MYXE_RS14505, MYXE_RS14565, MYXE_RS14600, MYXE_RS14650,

MYXE_RS14870, MYXE_RS14895, MYXE_RS14950, MYXE_RS14980, MYXE_RS15010,

MYXE_RS15145, MYXE_RS15200, MYXE_RS15215, MYXE_RS15510, MYXE_RS15550,

MYXE_RS15590, MYXE_RS15600, MYXE_RS15615, MYXE_RS15780, MYXE_RS15835,

MYXE_RS15840, MYXE_RS15935, MYXE_RS15945, MYXE_RS15975, MYXE_RS15980,

MYXE_RS16015, MYXE_RS16050, MYXE_RS16125, MYXE_RS16150, MYXE_RS16350,

MYXE_RS16430, MYXE_RS16590, MYXE_RS16595, MYXE_RS16610, MYXE_RS16630,

MYXE_RS16820, MYXE_RS16860, MYXE_RS17005, MYXE_RS17185, MYXE_RS17190,

MYXE_RS17195, MYXE_RS17215, MYXE_RS17320, MYXE_RS17335, MYXE_RS17400,

MYXE_RS17440, MYXE_RS17550, MYXE_RS17825, MYXE_RS17830, MYXE_RS18110,

MYXE_RS18120, MYXE_RS18130, MYXE_RS18275, MYXE_RS18285, MYXE_RS18290,

MYXE_RS18330, MYXE_RS18395, MYXE_RS18425, MYXE_RS18430, MYXE_RS18455,

MYXE_RS18485, MYXE_RS18625, MYXE_RS18690, MYXE_RS18695, MYXE_RS18715,

MYXE_RS18760, MYXE_RS18875, MYXE_RS18915, MYXE_RS19035, MYXE_RS19040,

MYXE_RS19175, MYXE_RS19190, MYXE_RS19550, MYXE_RS19705, MYXE_RS19730,

MYXE_RS19735, MYXE_RS19805, MYXE_RS19885, MYXE_RS19930, MYXE_RS19965,

MYXE_RS19975, MYXE_RS20130, MYXE_RS20210, MYXE_RS20410, MYXE_RS20435,

MYXE_RS20505, MYXE_RS20570, MYXE_RS20590, MYXE_RS20660, MYXE_RS20690,

MYXE_RS20715, MYXE_RS20725, MYXE_RS20770, MYXE_RS20800, MYXE_RS20830,

MYXE_RS20875, MYXE_RS20940, MYXE_RS20960, MYXE_RS21260, MYXE_RS21355,

MYXE_RS21400, MYXE_RS21490, MYXE_RS21530, MYXE_RS21740, MYXE_RS21790,

MYXE_RS21945, MYXE_RS21995, MYXE_RS22055, MYXE_RS22060, MYXE_RS22180,

MYXE_RS22215, MYXE_RS22465, MYXE_RS22470, MYXE_RS22520, MYXE_RS22550,

MYXE_RS22560, MYXE_RS22640, MYXE_RS22830, MYXE_RS22870, MYXE_RS22905,

MYXE_RS22925, MYXE_RS22955, MYXE_RS23010, MYXE_RS23220, MYXE_RS23250,

MYXE_RS23285, MYXE_RS23455, MYXE_RS23515, MYXE_RS23740, MYXE_RS23855,

MYXE_RS23940, MYXE_RS24010, MYXE_RS24100, MYXE_RS24110, MYXE_RS24185,

MYXE_RS24195, MYXE_RS24265, MYXE_RS24300, MYXE_RS24335, MYXE_RS24350,

MYXE_RS24355, MYXE_RS24450, MYXE_RS24460, MYXE_RS24575, MYXE_RS24595,

MYXE_RS24600, MYXE_RS24610, MYXE_RS24650, MYXE_RS24665, MYXE_RS24675,

MYXE_RS24755, MYXE_RS24760, MYXE_RS24770, MYXE_RS24790, MYXE_RS24835,

MYXE_RS24900, MYXE_RS24905, MYXE_RS24995, MYXE_RS25020, MYXE_RS25050,

MYXE_RS25055, MYXE_RS25070, MYXE_RS25080, MYXE_RS25085, MYXE_RS25135,

MYXE_RS25145, MYXE_RS25150, MYXE_RS25180, MYXE_RS25190, MYXE_RS25220,

MYXE_RS25230, MYXE_RS25260, MYXE_RS25265, MYXE_RS25270, MYXE_RS25290,

MYXE_RS25300, MYXE_RS25335, MYXE_RS25340, MYXE_RS25345, MYXE_RS25350,

MYXE_RS25360, MYXE_RS25365, MYXE_RS25380, MYXE_RS25385, MYXE_RS25390,

MYXE_RS25400

**Seed Genome Homologous Gene Filter**: filtered out 117 targets (discarded)

MYXE_RS00450, MYXE_RS00690, MYXE_RS01160, MYXE_RS01520, MYXE_RS01815,

MYXE_RS01925, MYXE_RS02465, MYXE_RS02490, MYXE_RS02720, MYXE_RS02725,

MYXE_RS02730, MYXE_RS02740, MYXE_RS03465, MYXE_RS03680, MYXE_RS03695,

MYXE_RS03700, MYXE_RS03980, MYXE_RS03985, MYXE_RS04780, MYXE_RS05020,

MYXE_RS05885, MYXE_RS06455, MYXE_RS06460, MYXE_RS06815, MYXE_RS06825,

MYXE_RS06830, MYXE_RS06835, MYXE_RS06840, MYXE_RS06845, MYXE_RS06875,

MYXE_RS07105, MYXE_RS07880, MYXE_RS07890, MYXE_RS07940, MYXE_RS08015,

MYXE_RS08020, MYXE_RS08555, MYXE_RS09030, MYXE_RS09695, MYXE_RS09700,

MYXE_RS09705, MYXE_RS09710, MYXE_RS09775, MYXE_RS10120, MYXE_RS10655,

MYXE_RS10660, MYXE_RS10670, MYXE_RS10965, MYXE_RS11545, MYXE_RS11800,

MYXE_RS12065, MYXE_RS12510, MYXE_RS12520, MYXE_RS12575, MYXE_RS12580,

MYXE_RS12615, MYXE_RS14540, MYXE_RS15190, MYXE_RS15205, MYXE_RS15845,

MYXE_RS16900, MYXE_RS16945, MYXE_RS17210, MYXE_RS17220, MYXE_RS17225,

MYXE_RS17230, MYXE_RS18035, MYXE_RS18040, MYXE_RS18090, MYXE_RS18445,

MYXE_RS18565, MYXE_RS19580, MYXE_RS19585, MYXE_RS19630, MYXE_RS19635,

MYXE_RS19665, MYXE_RS19670, MYXE_RS19680, MYXE_RS20430, MYXE_RS20535,

MYXE_RS20565, MYXE_RS21100, MYXE_RS21845, MYXE_RS21850, MYXE_RS21875,

MYXE_RS21880, MYXE_RS21885, MYXE_RS21890, MYXE_RS21955, MYXE_RS21960,

MYXE_RS21970, MYXE_RS21975, MYXE_RS21980, MYXE_RS22010, MYXE_RS22015,

MYXE_RS23350, MYXE_RS23835, MYXE_RS23930, MYXE_RS24320, MYXE_RS24485,

MYXE_RS24655, MYXE_RS24720, MYXE_RS24775, MYXE_RS24780, MYXE_RS24800,

MYXE_RS24910, MYXE_RS24945, MYXE_RS24975, MYXE_RS25030, MYXE_RS25035,

MYXE_RS25040, MYXE_RS25045, MYXE_RS25075, MYXE_RS25100, MYXE_RS25125,

MYXE_RS25185, MYXE_RS25200

**Seed Genome Gene Overlap Filter**: filtered out 229 targets (moved to Accessory)

MYXE_RS00080, MYXE_RS00095, MYXE_RS00150, MYXE_RS00250, MYXE_RS00705,

MYXE_RS01095, MYXE_RS01100, MYXE_RS01150, MYXE_RS01330, MYXE_RS01410,

MYXE_RS01790, MYXE_RS01890, MYXE_RS01905, MYXE_RS01940, MYXE_RS02040,

MYXE_RS02110, MYXE_RS02165, MYXE_RS02215, MYXE_RS02290, MYXE_RS02295,

MYXE_RS02475, MYXE_RS02535, MYXE_RS02545, MYXE_RS02570, MYXE_RS02700,

MYXE_RS02795, MYXE_RS02875, MYXE_RS02890, MYXE_RS03080, MYXE_RS03135,

MYXE_RS03275, MYXE_RS03405, MYXE_RS03835, MYXE_RS04050, MYXE_RS04065,

MYXE_RS04155, MYXE_RS04345, MYXE_RS04580, MYXE_RS04690, MYXE_RS04795,

MYXE_RS04925, MYXE_RS05090, MYXE_RS05100, MYXE_RS05135, MYXE_RS05210,

MYXE_RS05245, MYXE_RS05790, MYXE_RS05850, MYXE_RS05930, MYXE_RS06030,

MYXE_RS06100, MYXE_RS06255, MYXE_RS06325, MYXE_RS06360, MYXE_RS06530,

MYXE_RS06690, MYXE_RS06715, MYXE_RS06770, MYXE_RS07170, MYXE_RS07300,

MYXE_RS07370, MYXE_RS07525, MYXE_RS07575, MYXE_RS07645, MYXE_RS07805,

MYXE_RS07950, MYXE_RS08090, MYXE_RS08100, MYXE_RS08105, MYXE_RS08230,

MYXE_RS08275, MYXE_RS08510, MYXE_RS08595, MYXE_RS08615, MYXE_RS08650,

MYXE_RS09085, MYXE_RS09195, MYXE_RS09370, MYXE_RS09385, MYXE_RS09395,

MYXE_RS09495, MYXE_RS09510, MYXE_RS09600, MYXE_RS09845, MYXE_RS09905,

MYXE_RS09920, MYXE_RS10020, MYXE_RS10060, MYXE_RS10090, MYXE_RS10395,

MYXE_RS10440, MYXE_RS10475, MYXE_RS10515, MYXE_RS10535, MYXE_RS10580,

MYXE_RS10690, MYXE_RS11015, MYXE_RS11270, MYXE_RS11340, MYXE_RS11375,

MYXE_RS11420, MYXE_RS11430, MYXE_RS11455, MYXE_RS11560, MYXE_RS11750,

MYXE_RS11935, MYXE_RS12130, MYXE_RS12185, MYXE_RS12210, MYXE_RS12355,

MYXE_RS12535, MYXE_RS12625, MYXE_RS12800, MYXE_RS12885, MYXE_RS13020,

MYXE_RS13070, MYXE_RS13175, MYXE_RS13210, MYXE_RS13235, MYXE_RS13430,

MYXE_RS13745, MYXE_RS14000, MYXE_RS14135, MYXE_RS14180, MYXE_RS14235,

MYXE_RS14460, MYXE_RS14490, MYXE_RS14530, MYXE_RS14580, MYXE_RS14775,

MYXE_RS14785, MYXE_RS14855, MYXE_RS14910, MYXE_RS15035, MYXE_RS15125,

MYXE_RS15180, MYXE_RS15340, MYXE_RS15360, MYXE_RS15440, MYXE_RS15540,

MYXE_RS16000, MYXE_RS16135, MYXE_RS16155, MYXE_RS16605, MYXE_RS16765,

MYXE_RS16990, MYXE_RS17030, MYXE_RS17250, MYXE_RS17270, MYXE_RS17300,

MYXE_RS17405, MYXE_RS17530, MYXE_RS17570, MYXE_RS17590, MYXE_RS17795,

MYXE_RS17815, MYXE_RS17860, MYXE_RS17875, MYXE_RS17890, MYXE_RS17935,

MYXE_RS17945, MYXE_RS17950, MYXE_RS17965, MYXE_RS18005, MYXE_RS18315,

MYXE_RS18360, MYXE_RS18385, MYXE_RS18495, MYXE_RS18530, MYXE_RS18605,

MYXE_RS18645, MYXE_RS18745, MYXE_RS18865, MYXE_RS18930, MYXE_RS18985,

MYXE_RS19205, MYXE_RS19215, MYXE_RS19370, MYXE_RS19415, MYXE_RS19470,

MYXE_RS19520, MYXE_RS19790, MYXE_RS19820, MYXE_RS19855, MYXE_RS20125,

MYXE_RS20245, MYXE_RS20300, MYXE_RS20665, MYXE_RS20730, MYXE_RS20785,

MYXE_RS20810, MYXE_RS20885, MYXE_RS20905, MYXE_RS20935, MYXE_RS21070,

MYXE_RS21195, MYXE_RS21210, MYXE_RS21235, MYXE_RS21275, MYXE_RS21460,

MYXE_RS21590, MYXE_RS21600, MYXE_RS21710, MYXE_RS22030, MYXE_RS22335,

MYXE_RS22705, MYXE_RS22725, MYXE_RS23245, MYXE_RS23395, MYXE_RS23550,

MYXE_RS23565, MYXE_RS23710, MYXE_RS23985, MYXE_RS24375, MYXE_RS24390,

MYXE_RS24535, MYXE_RS24550, MYXE_RS24660, MYXE_RS24680, MYXE_RS24815,

MYXE_RS25105, MYXE_RS25120, MYXE_RS25195, MYXE_RS25235, MYXE_RS25255,

MYXE_RS25275, MYXE_RS25295, MYXE_RS25305, MYXE_RS25370

**Query Genome BLAST Search:** filtered out 1339 targets (moved to Accessory)

MYXE_RS00020, MYXE_RS00030, MYXE_RS00055, MYXE_RS00070, MYXE_RS00090,

MYXE_RS00100, MYXE_RS00105, MYXE_RS00120, MYXE_RS00205, MYXE_RS00215,

MYXE_RS00230, MYXE_RS00245, MYXE_RS00270, MYXE_RS00335, MYXE_RS00345,

MYXE_RS00375, MYXE_RS00455, MYXE_RS00460, MYXE_RS00470, MYXE_RS00475,

MYXE_RS00480, MYXE_RS00485, MYXE_RS00490, MYXE_RS00495, MYXE_RS00500,

MYXE_RS00515, MYXE_RS00615, MYXE_RS00620, MYXE_RS00625, MYXE_RS00630,

MYXE_RS00635, MYXE_RS00640, MYXE_RS00645, MYXE_RS00650, MYXE_RS00655,

MYXE_RS00660, MYXE_RS00665, MYXE_RS00670, MYXE_RS00675, MYXE_RS00685,

MYXE_RS00700, MYXE_RS00710, MYXE_RS00715, MYXE_RS00720, MYXE_RS00725,

MYXE_RS00730, MYXE_RS00735, MYXE_RS00740, MYXE_RS00745, MYXE_RS00750,

MYXE_RS00755, MYXE_RS00760, MYXE_RS00765, MYXE_RS00770, MYXE_RS00775,

MYXE_RS00780, MYXE_RS00785, MYXE_RS00790, MYXE_RS00795, MYXE_RS00800,

MYXE_RS00805, MYXE_RS00810, MYXE_RS00820, MYXE_RS00825, MYXE_RS00830,

MYXE_RS00835, MYXE_RS00840, MYXE_RS00850, MYXE_RS00855, MYXE_RS00860,

MYXE_RS00865, MYXE_RS00870, MYXE_RS00875, MYXE_RS00905, MYXE_RS00910,

MYXE_RS00915, MYXE_RS00920, MYXE_RS00925, MYXE_RS00930, MYXE_RS00935,

MYXE_RS00940, MYXE_RS00945, MYXE_RS00950, MYXE_RS00955, MYXE_RS00970,

MYXE_RS00975, MYXE_RS01040, MYXE_RS01045, MYXE_RS01050, MYXE_RS01080,

MYXE_RS01140, MYXE_RS01180, MYXE_RS01250, MYXE_RS01255, MYXE_RS01265,

MYXE_RS01270, MYXE_RS01290, MYXE_RS01310, MYXE_RS01375, MYXE_RS01455,

MYXE_RS01460, MYXE_RS01465, MYXE_RS01470, MYXE_RS01485, MYXE_RS01495,

MYXE_RS01500, MYXE_RS01515, MYXE_RS01530, MYXE_RS01565, MYXE_RS01600,

MYXE_RS01660, MYXE_RS01675, MYXE_RS01695, MYXE_RS01705, MYXE_RS01725,

MYXE_RS01765, MYXE_RS01900, MYXE_RS01965, MYXE_RS01975, MYXE_RS02055,

MYXE_RS02060, MYXE_RS02090, MYXE_RS02145, MYXE_RS02175, MYXE_RS02265,

MYXE_RS02285, MYXE_RS02300, MYXE_RS02310, MYXE_RS02340, MYXE_RS02350,

MYXE_RS02360, MYXE_RS02395, MYXE_RS02415, MYXE_RS02470, MYXE_RS02480,

MYXE_RS02500, MYXE_RS02505, MYXE_RS02510, MYXE_RS02515, MYXE_RS02520,

MYXE_RS02525, MYXE_RS02530, MYXE_RS02540, MYXE_RS02550, MYXE_RS02555,

MYXE_RS02560, MYXE_RS02565, MYXE_RS02575, MYXE_RS02580, MYXE_RS02585,

MYXE_RS02590, MYXE_RS02595, MYXE_RS02600, MYXE_RS02605, MYXE_RS02610,

MYXE_RS02615, MYXE_RS02620, MYXE_RS02625, MYXE_RS02630, MYXE_RS02635,

MYXE_RS02640, MYXE_RS02645, MYXE_RS02650, MYXE_RS02655, MYXE_RS02660,

MYXE_RS02665, MYXE_RS02670, MYXE_RS02675, MYXE_RS02685, MYXE_RS02690,

MYXE_RS02695, MYXE_RS02705, MYXE_RS02710, MYXE_RS02715, MYXE_RS02750,

MYXE_RS02755, MYXE_RS02760, MYXE_RS02765, MYXE_RS02770, MYXE_RS02775,

MYXE_RS02780, MYXE_RS02785, MYXE_RS02790, MYXE_RS02800, MYXE_RS02805,

MYXE_RS02810, MYXE_RS02820, MYXE_RS02940, MYXE_RS02945, MYXE_RS03030,

MYXE_RS03050, MYXE_RS03065, MYXE_RS03150, MYXE_RS03235, MYXE_RS03255,

MYXE_RS03325, MYXE_RS03345, MYXE_RS03430, MYXE_RS03510, MYXE_RS03590,

MYXE_RS03600, MYXE_RS03610, MYXE_RS03650, MYXE_RS03685, MYXE_RS03690,

MYXE_RS03710, MYXE_RS03715, MYXE_RS03720, MYXE_RS03730, MYXE_RS03735,

MYXE_RS03740, MYXE_RS03745, MYXE_RS03750, MYXE_RS03755, MYXE_RS03760,

MYXE_RS03765, MYXE_RS03770, MYXE_RS03780, MYXE_RS03785, MYXE_RS03790,

MYXE_RS03795, MYXE_RS03825, MYXE_RS03830, MYXE_RS03840, MYXE_RS03845,

MYXE_RS03850, MYXE_RS03855, MYXE_RS03860, MYXE_RS03870, MYXE_RS03875,

MYXE_RS03885, MYXE_RS03890, MYXE_RS03895, MYXE_RS03900, MYXE_RS03905,

MYXE_RS03910, MYXE_RS03915, MYXE_RS03920, MYXE_RS03925, MYXE_RS03930,

MYXE_RS03935, MYXE_RS03940, MYXE_RS03945, MYXE_RS03950, MYXE_RS03955,

MYXE_RS03960, MYXE_RS03965, MYXE_RS03970, MYXE_RS03990, MYXE_RS03995,

MYXE_RS04000, MYXE_RS04010, MYXE_RS04015, MYXE_RS04020, MYXE_RS04025,

MYXE_RS04030, MYXE_RS04035, MYXE_RS04040, MYXE_RS04045, MYXE_RS04055,

MYXE_RS04060, MYXE_RS04070, MYXE_RS04075, MYXE_RS04080, MYXE_RS04085,

MYXE_RS04105, MYXE_RS04115, MYXE_RS04120, MYXE_RS04130, MYXE_RS04135,

MYXE_RS04140, MYXE_RS04145, MYXE_RS04150, MYXE_RS04160, MYXE_RS04165,

MYXE_RS04170, MYXE_RS04175, MYXE_RS04180, MYXE_RS04185, MYXE_RS04190,

MYXE_RS04195, MYXE_RS04200, MYXE_RS04205, MYXE_RS04210, MYXE_RS04215,

MYXE_RS04220, MYXE_RS04225, MYXE_RS04230, MYXE_RS04235, MYXE_RS04240,

MYXE_RS04245, MYXE_RS04250, MYXE_RS04255, MYXE_RS04260, MYXE_RS04265,

MYXE_RS04270, MYXE_RS04275, MYXE_RS04280, MYXE_RS04285, MYXE_RS04290,

MYXE_RS04295, MYXE_RS04300, MYXE_RS04305, MYXE_RS04310, MYXE_RS04320,

MYXE_RS04325, MYXE_RS04330, MYXE_RS04335, MYXE_RS04340, MYXE_RS04350,

MYXE_RS04355, MYXE_RS04360, MYXE_RS04365, MYXE_RS04380, MYXE_RS04385,

MYXE_RS04390, MYXE_RS04395, MYXE_RS04400, MYXE_RS04405, MYXE_RS04415,

MYXE_RS04420, MYXE_RS04430, MYXE_RS04435, MYXE_RS04440, MYXE_RS04445,

MYXE_RS04455, MYXE_RS04460, MYXE_RS04465, MYXE_RS04470, MYXE_RS04475,

MYXE_RS04480, MYXE_RS04485, MYXE_RS04500, MYXE_RS04520, MYXE_RS04525,

MYXE_RS04530, MYXE_RS04535, MYXE_RS04540, MYXE_RS04545, MYXE_RS04550,

MYXE_RS04560, MYXE_RS04565, MYXE_RS04570, MYXE_RS04575, MYXE_RS04585,

MYXE_RS04595, MYXE_RS04600, MYXE_RS04605, MYXE_RS04610, MYXE_RS04615,

MYXE_RS04620, MYXE_RS04785, MYXE_RS04800, MYXE_RS04850, MYXE_RS04870,

MYXE_RS04905, MYXE_RS04910, MYXE_RS04915, MYXE_RS04920, MYXE_RS04930,

MYXE_RS04935, MYXE_RS04940, MYXE_RS04945, MYXE_RS04950, MYXE_RS04955,

MYXE_RS04960, MYXE_RS04965, MYXE_RS04970, MYXE_RS04980, MYXE_RS04985,

MYXE_RS04990, MYXE_RS04995, MYXE_RS05000, MYXE_RS05005, MYXE_RS05010,

MYXE_RS05015, MYXE_RS05025, MYXE_RS05030, MYXE_RS05035, MYXE_RS05040,

MYXE_RS05045, MYXE_RS05055, MYXE_RS05060, MYXE_RS05145, MYXE_RS05175,

MYXE_RS05200, MYXE_RS05250, MYXE_RS05295, MYXE_RS05320, MYXE_RS05335,

MYXE_RS05340, MYXE_RS05345, MYXE_RS05355, MYXE_RS05395, MYXE_RS05400,

MYXE_RS05405, MYXE_RS05450, MYXE_RS05455, MYXE_RS05520, MYXE_RS05550,

MYXE_RS05575, MYXE_RS05665, MYXE_RS05705, MYXE_RS05750, MYXE_RS05765,

MYXE_RS05775, MYXE_RS05830, MYXE_RS05840, MYXE_RS05875, MYXE_RS05895,

MYXE_RS05910, MYXE_RS05955, MYXE_RS06005, MYXE_RS06015, MYXE_RS06020,

MYXE_RS06045, MYXE_RS06085, MYXE_RS06115, MYXE_RS06165, MYXE_RS06170,

MYXE_RS06180, MYXE_RS06185, MYXE_RS06200, MYXE_RS06205, MYXE_RS06220,

MYXE_RS06235, MYXE_RS06335, MYXE_RS06340, MYXE_RS06355, MYXE_RS06390,

MYXE_RS06420, MYXE_RS06425, MYXE_RS06430, MYXE_RS06435, MYXE_RS06450,

MYXE_RS06465, MYXE_RS06475, MYXE_RS06570, MYXE_RS06615, MYXE_RS06620,

MYXE_RS06625, MYXE_RS06630, MYXE_RS06675, MYXE_RS06720, MYXE_RS06740,

MYXE_RS06805, MYXE_RS06865, MYXE_RS06880, MYXE_RS06905, MYXE_RS06985,

MYXE_RS07010, MYXE_RS07040, MYXE_RS07050, MYXE_RS07055, MYXE_RS07085,

MYXE_RS07090, MYXE_RS07095, MYXE_RS07110, MYXE_RS07115, MYXE_RS07120,

MYXE_RS07130, MYXE_RS07145, MYXE_RS07150, MYXE_RS07155, MYXE_RS07160,

MYXE_RS07165, MYXE_RS07175, MYXE_RS07180, MYXE_RS07205, MYXE_RS07210,

MYXE_RS07215, MYXE_RS07220, MYXE_RS07225, MYXE_RS07235, MYXE_RS07240,

MYXE_RS07245, MYXE_RS07250, MYXE_RS07255, MYXE_RS07260, MYXE_RS07265,

MYXE_RS07275, MYXE_RS07280, MYXE_RS07290, MYXE_RS07295, MYXE_RS07305,

MYXE_RS07315, MYXE_RS07320, MYXE_RS07330, MYXE_RS07335, MYXE_RS07365,

MYXE_RS07455, MYXE_RS07490, MYXE_RS07500, MYXE_RS07505, MYXE_RS07540,

MYXE_RS07545, MYXE_RS07565, MYXE_RS07640, MYXE_RS07650, MYXE_RS07690,

MYXE_RS07710, MYXE_RS07715, MYXE_RS07720, MYXE_RS07725, MYXE_RS07735,

MYXE_RS07825, MYXE_RS07845, MYXE_RS07955, MYXE_RS08040, MYXE_RS08050,

MYXE_RS08065, MYXE_RS08070, MYXE_RS08200, MYXE_RS08215, MYXE_RS08250,

MYXE_RS08255, MYXE_RS08300, MYXE_RS08350, MYXE_RS08365, MYXE_RS08390,

MYXE_RS08425, MYXE_RS08470, MYXE_RS08485, MYXE_RS08525, MYXE_RS08575,

MYXE_RS08590, MYXE_RS08750, MYXE_RS08755, MYXE_RS08770, MYXE_RS08780,

MYXE_RS08810, MYXE_RS08820, MYXE_RS08825, MYXE_RS08830, MYXE_RS08895,

MYXE_RS08940, MYXE_RS08945, MYXE_RS08955, MYXE_RS08960, MYXE_RS08965,

MYXE_RS09000, MYXE_RS09015, MYXE_RS09020, MYXE_RS09040, MYXE_RS09055,

MYXE_RS09065, MYXE_RS09105, MYXE_RS09225, MYXE_RS09260, MYXE_RS09265,

MYXE_RS09270, MYXE_RS09275, MYXE_RS09280, MYXE_RS09290, MYXE_RS09295,

MYXE_RS09300, MYXE_RS09305, MYXE_RS09310, MYXE_RS09315, MYXE_RS09320,

MYXE_RS09325, MYXE_RS09330, MYXE_RS09335, MYXE_RS09340, MYXE_RS09345,

MYXE_RS09350, MYXE_RS09355, MYXE_RS09360, MYXE_RS09375, MYXE_RS09380,

MYXE_RS09390, MYXE_RS09400, MYXE_RS09405, MYXE_RS09410, MYXE_RS09415,

MYXE_RS09420, MYXE_RS09430, MYXE_RS09440, MYXE_RS09450, MYXE_RS09455,

MYXE_RS09460, MYXE_RS09465, MYXE_RS09470, MYXE_RS09475, MYXE_RS09480,

MYXE_RS09490, MYXE_RS09505, MYXE_RS09515, MYXE_RS09520, MYXE_RS09525,

MYXE_RS09530, MYXE_RS09535, MYXE_RS09550, MYXE_RS09555, MYXE_RS09565,

MYXE_RS09570, MYXE_RS09575, MYXE_RS09580, MYXE_RS09585, MYXE_RS09590,

MYXE_RS09595, MYXE_RS09625, MYXE_RS09630, MYXE_RS09635, MYXE_RS09645,

MYXE_RS09650, MYXE_RS09655, MYXE_RS09660, MYXE_RS09665, MYXE_RS09670,

MYXE_RS09680, MYXE_RS09685, MYXE_RS09715, MYXE_RS09730, MYXE_RS09790,

MYXE_RS09795, MYXE_RS09805, MYXE_RS09895, MYXE_RS09915, MYXE_RS09925,

MYXE_RS09930, MYXE_RS09965, MYXE_RS10040, MYXE_RS10045, MYXE_RS10080,

MYXE_RS10085, MYXE_RS10110, MYXE_RS10145, MYXE_RS10160, MYXE_RS10190,

MYXE_RS10225, MYXE_RS10230, MYXE_RS10260, MYXE_RS10305, MYXE_RS10320,

MYXE_RS10330, MYXE_RS10335, MYXE_RS10355, MYXE_RS10375, MYXE_RS10380,

MYXE_RS10405, MYXE_RS10435, MYXE_RS10500, MYXE_RS10520, MYXE_RS10525,

MYXE_RS10540, MYXE_RS10565, MYXE_RS10575, MYXE_RS10595, MYXE_RS10625,

MYXE_RS10630, MYXE_RS10635, MYXE_RS10640, MYXE_RS10645, MYXE_RS10650,

MYXE_RS10675, MYXE_RS10680, MYXE_RS10685, MYXE_RS10695, MYXE_RS10700,

MYXE_RS10715, MYXE_RS10720, MYXE_RS10725, MYXE_RS10730, MYXE_RS10735,

MYXE_RS10740, MYXE_RS10745, MYXE_RS10750, MYXE_RS10755, MYXE_RS10760,

MYXE_RS10765, MYXE_RS10770, MYXE_RS10775, MYXE_RS10780, MYXE_RS10790,

MYXE_RS10795, MYXE_RS10805, MYXE_RS10810, MYXE_RS10815, MYXE_RS10825,

MYXE_RS10830, MYXE_RS10835, MYXE_RS10840, MYXE_RS10845, MYXE_RS10855,

MYXE_RS10860, MYXE_RS10865, MYXE_RS10875, MYXE_RS10880, MYXE_RS10885,

MYXE_RS10890, MYXE_RS10895, MYXE_RS10900, MYXE_RS10905, MYXE_RS10910,

MYXE_RS10915, MYXE_RS10925, MYXE_RS10930, MYXE_RS10935, MYXE_RS10940,

MYXE_RS10945, MYXE_RS10950, MYXE_RS10955, MYXE_RS10960, MYXE_RS10980,

MYXE_RS10995, MYXE_RS11050, MYXE_RS11055, MYXE_RS11060, MYXE_RS11065,

MYXE_RS11070, MYXE_RS11080, MYXE_RS11085, MYXE_RS11090, MYXE_RS11095,

MYXE_RS11100, MYXE_RS11125, MYXE_RS11135, MYXE_RS11200, MYXE_RS11210,

MYXE_RS11215, MYXE_RS11220, MYXE_RS11245, MYXE_RS11265, MYXE_RS11280,

MYXE_RS11295, MYXE_RS11395, MYXE_RS11425, MYXE_RS11435, MYXE_RS11500,

MYXE_RS11505, MYXE_RS11515, MYXE_RS11520, MYXE_RS11525, MYXE_RS11530,

MYXE_RS11535, MYXE_RS11580, MYXE_RS11585, MYXE_RS11590, MYXE_RS11610,

MYXE_RS11620, MYXE_RS11630, MYXE_RS11680, MYXE_RS11695, MYXE_RS11700,

MYXE_RS11715, MYXE_RS11720, MYXE_RS11725, MYXE_RS11730, MYXE_RS11735,

MYXE_RS11740, MYXE_RS11745, MYXE_RS11820, MYXE_RS11870, MYXE_RS11905,

MYXE_RS11925, MYXE_RS11950, MYXE_RS11955, MYXE_RS11985, MYXE_RS12035,

MYXE_RS12045, MYXE_RS12060, MYXE_RS12070, MYXE_RS12105, MYXE_RS12115,

MYXE_RS12125, MYXE_RS12190, MYXE_RS12200, MYXE_RS12205, MYXE_RS12240,

MYXE_RS12280, MYXE_RS12340, MYXE_RS12345, MYXE_RS12365, MYXE_RS12430,

MYXE_RS12440, MYXE_RS12470, MYXE_RS12475, MYXE_RS12480, MYXE_RS12485,

MYXE_RS12490, MYXE_RS12495, MYXE_RS12515, MYXE_RS12540, MYXE_RS12545,

MYXE_RS12550, MYXE_RS12585, MYXE_RS12595, MYXE_RS12605, MYXE_RS12620,

MYXE_RS12630, MYXE_RS12635, MYXE_RS12640, MYXE_RS12660, MYXE_RS12680,

MYXE_RS12730, MYXE_RS12735, MYXE_RS12745, MYXE_RS12820, MYXE_RS12890,

MYXE_RS12925, MYXE_RS12980, MYXE_RS13000, MYXE_RS13015, MYXE_RS13040,

MYXE_RS13045, MYXE_RS13075, MYXE_RS13085, MYXE_RS13095, MYXE_RS13100,

MYXE_RS13105, MYXE_RS13110, MYXE_RS13115, MYXE_RS13120, MYXE_RS13125,

MYXE_RS13130, MYXE_RS13140, MYXE_RS13160, MYXE_RS13165, MYXE_RS13185,

MYXE_RS13305, MYXE_RS13335, MYXE_RS13345, MYXE_RS13350, MYXE_RS13395,

MYXE_RS13425, MYXE_RS13470, MYXE_RS13475, MYXE_RS13480, MYXE_RS13500,

MYXE_RS13510, MYXE_RS13525, MYXE_RS13590, MYXE_RS13605, MYXE_RS13625,

MYXE_RS13670, MYXE_RS13680, MYXE_RS13710, MYXE_RS13720, MYXE_RS13770,

MYXE_RS13785, MYXE_RS13820, MYXE_RS13855, MYXE_RS13950, MYXE_RS14010,

MYXE_RS14060, MYXE_RS14065, MYXE_RS14085, MYXE_RS14090, MYXE_RS14130,

MYXE_RS14160, MYXE_RS14165, MYXE_RS14290, MYXE_RS14300, MYXE_RS14320,

MYXE_RS14335, MYXE_RS14360, MYXE_RS14365, MYXE_RS14370, MYXE_RS14395,

MYXE_RS14415, MYXE_RS14420, MYXE_RS14430, MYXE_RS14445, MYXE_RS14465,

MYXE_RS14510, MYXE_RS14515, MYXE_RS14520, MYXE_RS14525, MYXE_RS14535,

MYXE_RS14545, MYXE_RS14550, MYXE_RS14555, MYXE_RS14560, MYXE_RS14570,

MYXE_RS14605, MYXE_RS14635, MYXE_RS14645, MYXE_RS14675, MYXE_RS14705,

MYXE_RS14730, MYXE_RS14735, MYXE_RS14740, MYXE_RS14745, MYXE_RS14755,

MYXE_RS14765, MYXE_RS14770, MYXE_RS14780, MYXE_RS14790, MYXE_RS14795,

MYXE_RS14800, MYXE_RS14810, MYXE_RS14815, MYXE_RS14825, MYXE_RS14875,

MYXE_RS14885, MYXE_RS14960, MYXE_RS15030, MYXE_RS15045, MYXE_RS15065,

MYXE_RS15070, MYXE_RS15090, MYXE_RS15100, MYXE_RS15110, MYXE_RS15115,

MYXE_RS15140, MYXE_RS15150, MYXE_RS15270, MYXE_RS15280, MYXE_RS15290,

MYXE_RS15350, MYXE_RS15365, MYXE_RS15390, MYXE_RS15395, MYXE_RS15400,

MYXE_RS15405, MYXE_RS15415, MYXE_RS15425, MYXE_RS15450, MYXE_RS15470,

MYXE_RS15480, MYXE_RS15490, MYXE_RS15500, MYXE_RS15525, MYXE_RS15580,

MYXE_RS15605, MYXE_RS15610, MYXE_RS15630, MYXE_RS15695, MYXE_RS15710,

MYXE_RS15725, MYXE_RS15730, MYXE_RS15795, MYXE_RS15800, MYXE_RS15860,

MYXE_RS15865, MYXE_RS15885, MYXE_RS15890, MYXE_RS15895, MYXE_RS15915,

MYXE_RS15950, MYXE_RS15960, MYXE_RS15985, MYXE_RS15990, MYXE_RS16005,

MYXE_RS16020, MYXE_RS16095, MYXE_RS16100, MYXE_RS16130, MYXE_RS16140,

MYXE_RS16185, MYXE_RS16275, MYXE_RS16390, MYXE_RS16400, MYXE_RS16420,

MYXE_RS16455, MYXE_RS16460, MYXE_RS16475, MYXE_RS16500, MYXE_RS16620,

MYXE_RS16645, MYXE_RS16675, MYXE_RS16680, MYXE_RS16685, MYXE_RS16690,

MYXE_RS16705, MYXE_RS16710, MYXE_RS16715, MYXE_RS16855, MYXE_RS16905,

MYXE_RS16920, MYXE_RS16930, MYXE_RS16935, MYXE_RS16985, MYXE_RS17010,

MYXE_RS17020, MYXE_RS17055, MYXE_RS17075, MYXE_RS17085, MYXE_RS17105,

MYXE_RS17110, MYXE_RS17120, MYXE_RS17155, MYXE_RS17160, MYXE_RS17175,

MYXE_RS17235, MYXE_RS17240, MYXE_RS17285, MYXE_RS17360, MYXE_RS17375,

MYXE_RS17430, MYXE_RS17435, MYXE_RS17455, MYXE_RS17465, MYXE_RS17470,

MYXE_RS17475, MYXE_RS17585, MYXE_RS17615, MYXE_RS17620, MYXE_RS17635,

MYXE_RS17650, MYXE_RS17685, MYXE_RS17690, MYXE_RS17715, MYXE_RS17725,

MYXE_RS17730, MYXE_RS17735, MYXE_RS17750, MYXE_RS17765, MYXE_RS17880,

MYXE_RS17910, MYXE_RS17920, MYXE_RS17930, MYXE_RS17990, MYXE_RS18030,

MYXE_RS18045, MYXE_RS18055, MYXE_RS18085, MYXE_RS18100, MYXE_RS18105,

MYXE_RS18140, MYXE_RS18165, MYXE_RS18170, MYXE_RS18185, MYXE_RS18210,

MYXE_RS18225, MYXE_RS18325, MYXE_RS18335, MYXE_RS18375, MYXE_RS18415,

MYXE_RS18470, MYXE_RS18480, MYXE_RS18560, MYXE_RS18570, MYXE_RS18620,

MYXE_RS18665, MYXE_RS18700, MYXE_RS18705, MYXE_RS18710, MYXE_RS18730,

MYXE_RS18775, MYXE_RS18830, MYXE_RS18845, MYXE_RS18855, MYXE_RS18890,

MYXE_RS18900, MYXE_RS18920, MYXE_RS18935, MYXE_RS18940, MYXE_RS18950,

MYXE_RS18995, MYXE_RS19010, MYXE_RS19025, MYXE_RS19100, MYXE_RS19195,

MYXE_RS19200, MYXE_RS19265, MYXE_RS19310, MYXE_RS19315, MYXE_RS19340,

MYXE_RS19345, MYXE_RS19355, MYXE_RS19380, MYXE_RS19385, MYXE_RS19390,

MYXE_RS19400, MYXE_RS19405, MYXE_RS19420, MYXE_RS19495, MYXE_RS19610,

MYXE_RS19615, MYXE_RS19620, MYXE_RS19625, MYXE_RS19640, MYXE_RS19645,

MYXE_RS19650, MYXE_RS19660, MYXE_RS19685, MYXE_RS19690, MYXE_RS19695,

MYXE_RS19700, MYXE_RS19710, MYXE_RS19715, MYXE_RS19725, MYXE_RS19745,

MYXE_RS19750, MYXE_RS19755, MYXE_RS19760, MYXE_RS19765, MYXE_RS19785,

MYXE_RS19945, MYXE_RS19950, MYXE_RS20010, MYXE_RS20070, MYXE_RS20085,

MYXE_RS20095, MYXE_RS20165, MYXE_RS20190, MYXE_RS20215, MYXE_RS20225,

MYXE_RS20235, MYXE_RS20280, MYXE_RS20295, MYXE_RS20370, MYXE_RS20385,

MYXE_RS20390, MYXE_RS20395, MYXE_RS20400, MYXE_RS20405, MYXE_RS20415,

MYXE_RS20420, MYXE_RS20440, MYXE_RS20445, MYXE_RS20450, MYXE_RS20455,

MYXE_RS20460, MYXE_RS20465, MYXE_RS20470, MYXE_RS20475, MYXE_RS20480,

MYXE_RS20485, MYXE_RS20490, MYXE_RS20495, MYXE_RS20500, MYXE_RS20510,

MYXE_RS20515, MYXE_RS20520, MYXE_RS20525, MYXE_RS20530, MYXE_RS20540,

MYXE_RS20545, MYXE_RS20550, MYXE_RS20555, MYXE_RS20575, MYXE_RS20580,

MYXE_RS20585, MYXE_RS20595, MYXE_RS20600, MYXE_RS20605, MYXE_RS20610,

MYXE_RS20615, MYXE_RS20620, MYXE_RS20625, MYXE_RS20630, MYXE_RS20655,

MYXE_RS20815, MYXE_RS20835, MYXE_RS20840, MYXE_RS20870, MYXE_RS20890,

MYXE_RS20910, MYXE_RS20915, MYXE_RS20920, MYXE_RS20975, MYXE_RS20980,

MYXE_RS21065, MYXE_RS21090, MYXE_RS21095, MYXE_RS21105, MYXE_RS21125,

MYXE_RS21135, MYXE_RS21140, MYXE_RS21145, MYXE_RS21150, MYXE_RS21155,

MYXE_RS21165, MYXE_RS21170, MYXE_RS21175, MYXE_RS21180, MYXE_RS21185,

MYXE_RS21245, MYXE_RS21255, MYXE_RS21265, MYXE_RS21270, MYXE_RS21305,

MYXE_RS21310, MYXE_RS21315, MYXE_RS21320, MYXE_RS21325, MYXE_RS21330,

MYXE_RS21335, MYXE_RS21340, MYXE_RS21345, MYXE_RS21440, MYXE_RS21445,

MYXE_RS21505, MYXE_RS21510, MYXE_RS21555, MYXE_RS21655, MYXE_RS21665,

MYXE_RS21685, MYXE_RS21690, MYXE_RS21695, MYXE_RS21700, MYXE_RS21705,

MYXE_RS21715, MYXE_RS21720, MYXE_RS21725, MYXE_RS21730, MYXE_RS21780,

MYXE_RS21785, MYXE_RS21795, MYXE_RS21810, MYXE_RS21855, MYXE_RS21860,

MYXE_RS21865, MYXE_RS21870, MYXE_RS21895, MYXE_RS21900, MYXE_RS21905,

MYXE_RS21925, MYXE_RS21930, MYXE_RS21940, MYXE_RS21965, MYXE_RS21985,

MYXE_RS21990, MYXE_RS22000, MYXE_RS22005, MYXE_RS22020, MYXE_RS22025,

MYXE_RS22065, MYXE_RS22120, MYXE_RS22140, MYXE_RS22175, MYXE_RS22190,

MYXE_RS22210, MYXE_RS22225, MYXE_RS22240, MYXE_RS22250, MYXE_RS22280,

MYXE_RS22285, MYXE_RS22365, MYXE_RS22420, MYXE_RS22455, MYXE_RS22475,

MYXE_RS22495, MYXE_RS22505, MYXE_RS22530, MYXE_RS22535, MYXE_RS22580,

MYXE_RS22585, MYXE_RS22590, MYXE_RS22600, MYXE_RS22660, MYXE_RS22680,

MYXE_RS22710, MYXE_RS22715, MYXE_RS22730, MYXE_RS22735, MYXE_RS22745,

MYXE_RS22890, MYXE_RS22970, MYXE_RS22995, MYXE_RS23000, MYXE_RS23040,

MYXE_RS23085, MYXE_RS23100, MYXE_RS23105, MYXE_RS23130, MYXE_RS23145,

MYXE_RS23155, MYXE_RS23175, MYXE_RS23205, MYXE_RS23295, MYXE_RS23320,

MYXE_RS23335, MYXE_RS23340, MYXE_RS23345, MYXE_RS23365, MYXE_RS23385,

MYXE_RS23390, MYXE_RS23400, MYXE_RS23415, MYXE_RS23435, MYXE_RS23495,

MYXE_RS23500, MYXE_RS23505, MYXE_RS23530, MYXE_RS23580, MYXE_RS23595,

MYXE_RS23600, MYXE_RS23615, MYXE_RS23620, MYXE_RS23625, MYXE_RS23660,

MYXE_RS23750, MYXE_RS23915, MYXE_RS23935, MYXE_RS23960, MYXE_RS24025,

MYXE_RS24045, MYXE_RS24055, MYXE_RS24105, MYXE_RS24140, MYXE_RS24180,

MYXE_RS24190, MYXE_RS24205, MYXE_RS24215, MYXE_RS24220, MYXE_RS24225,

MYXE_RS24315, MYXE_RS24325, MYXE_RS24330, MYXE_RS24365, MYXE_RS24370,

MYXE_RS24395, MYXE_RS24400, MYXE_RS24405, MYXE_RS24410, MYXE_RS24435,

MYXE_RS24440, MYXE_RS24445, MYXE_RS24605, MYXE_RS24615, MYXE_RS24640,

MYXE_RS24725, MYXE_RS24795, MYXE_RS24925, MYXE_RS24930, MYXE_RS24935,

MYXE_RS24940, MYXE_RS24955, MYXE_RS24960, MYXE_RS24965, MYXE_RS24970,

MYXE_RS24980, MYXE_RS24990, MYXE_RS25090, MYXE_RS25130, MYXE_RS25140,

MYXE_RS25155, MYXE_RS25165, MYXE_RS25215, MYXE_RS25250, MYXE_RS25280,

MYXE_RS25285, MYXE_RS25310, MYXE_RS25325, MYXE_RS25395

**Penetration Query Genomes Stop Codon Percentage Filter:** filtered out 14 targets (moved to Accessory)

MYXE_RS01990, MYXE_RS03370, MYXE_RS07600, MYXE_RS08935, MYXE_RS10530,

MYXE_RS11810, MYXE_RS12110, MYXE_RS13320, MYXE_RS16785, MYXE_RS17535,

MYXE_RS20845, MYXE_RS22985, MYXE_RS24700, MYXE_RS25315

Messages for NZ_AP022314.1 (13-FEB-2025), 4917655 bases, 4716 genes with CDS (Mycobacterium xenopi strain JCM 15661 chromosome, complete genome):

* Skipped MYXE_RS00005 because location is not contiguous.

### Settings for allele calling in SeqSphere+

If (new) assemblies are analysed with the newly created cgMLST scheme, a value called „**% of good cgMLST** targets“ is calculated by SeqSphere+. The % of good cgMLST targets is the percentage of total genes of the cgMLST scheme (in this case 2565 genes) that were assigned an allele number (i.e. were found in the assembly, and fulfilled all quality criteria as mentioned below).

**Good targets**: by default allele numbers are only assigned to a target if (https://www.ridom.de/seqsphere/ug/v90/General_Procedure.html):

1. **Only one homologues gene (BLAST hit) is found** within the query assembly (using BLASTN version 2.2.12 with word size: 11, mismatch penalty: -1, match reward: 1, gap open costs: 5, and gap extension costs: 2 using the scheme’s target sequences of the seed genome (ref.-seqs.) as query sequences and the to be compared genome contig(s) as subject sequence(s)). If multiple top-matching hits are found then no hit is selected to avoid ambiguous results.
2. The (extended) BLAST hit (i.e. target consensus sequence of the gene under consideration) has at **least 90% identity and 100% alignment** with the corresponding reference sequence of that target
3. The length of the (extended) BLAST hit equals the **reference sequence length** of that target **plus or minus three triplets**
4. There are **no ambiguous bases** (e.g. N) in the (extended) BLAST hit
5. There are **no frame shifts** in the (extended) BLAST hit compared to the targets`reference allele (i.e. the number of insertion(s) of the target consensus sequence in comparison to the reference sequence minus the number of deletion(s) must be dividable by 3)
6. An appropriate **start** (ATG, CTG, GTG or TTG) and **stop** codon is found and **no** **internal** **stop codon** was found

Note: a **missing target** can either be „not found (no blast hit)“ or „failed“ (not fulfilling point 3-6)

## Selection of threshold for recent transmission

Thresholds for defining recent transmission are typically established using epidemiologically related samples, such as multiple genomes from the same (type) strain, isolates from the same patient, or genomes from confirmed outbreak clusters. Because no outbreak-related whole genome sequencing data exist for M. xenopi, we relied on the limited within-patient isolate pairs from this study and on multiple publicly available genomes of the type strain to guide and validate our threshold choice. Note that different (yet plausible) thresholds can produce slightly different cluster compositions, particularly at low genetic distances. Therefore, thresholds should be viewed as practical guides that help steer epidemiological investigations or help to understand underlying phylogenies, rather than being definitive or biologically absolute cutoffs.

### Analysis of type strain genomes

The *M. xenopi* type strain was originally isolated from skin lesions found on a toad (Xenopus laevis) in 1959^2^. At the time of writing, three different genome datasets of the type strain were available ( We performed cgMLST analysis on all three available NCBI assemblies. In addition, we simulated sequencing reads from GCA_009936235.1 (JCM 15661) using dwgsim, reassembled them with shovill v1.1.0 with SKESA v2.4.0 as the assembler (designated GCA0099362351-JCM15661_skesa), and applied cgMLST to this dataset as well.

Table 1). These genomes were sequenced and assembled by different institutions with different assembly methods. We performed cgMLST analysis on all three available NCBI assemblies. In addition, we simulated sequencing reads from GCA_009936235.1 (JCM 15661) using dwgsim, reassembled them with shovill v1.1.0 with SKESA v2.4.0 as the assembler (designated GCA0099362351-JCM15661_skesa), and applied cgMLST to this dataset as well.

Table 1 Characteristics of available genome assemblies of the M. xenopi type strain in NCBI.

| **Type strain** | **Size**  **Mb** | **Contigs** | **Level** | **Release date** | **Submitter** | **Genes** | **CheckM completeness/ contamination %** |
| --- | --- | --- | --- | --- | --- | --- | --- |
| NCTC 10042  GCA_900453395.1 | 5.252 | 21 | Contig | 2018 | SC | 5118 | 98.0/1.41 |
| JCM 15661  GCA_009936235.1 | 4.918 | 1 | Complete | 2019 | National Institute of Infectious Diseases | 4771 | 91.41/0.93 |
| DSM 43995  GCA_002102015.1 | 4.933 | 146 | Contig | 2017 | University of Trento | 4858 | 98.0/2.08 |

No allele differences were observed between the original complete genome (GCA_009936235.1) and its SKESA reassembly (Figure SM2). The allele distance between the two other draft assemblies, GCA_900453395.1 and GCA_002102015.1, was also low (3 alleles). In contrast, these draft assemblies differed more substantially from the complete JCM 15661 genome (14 alleles), potentially reflecting genetic divergence due to long-term independent subculturing across strain collections, or alternatively, minor quality issues in one or more assemblies or core genes.


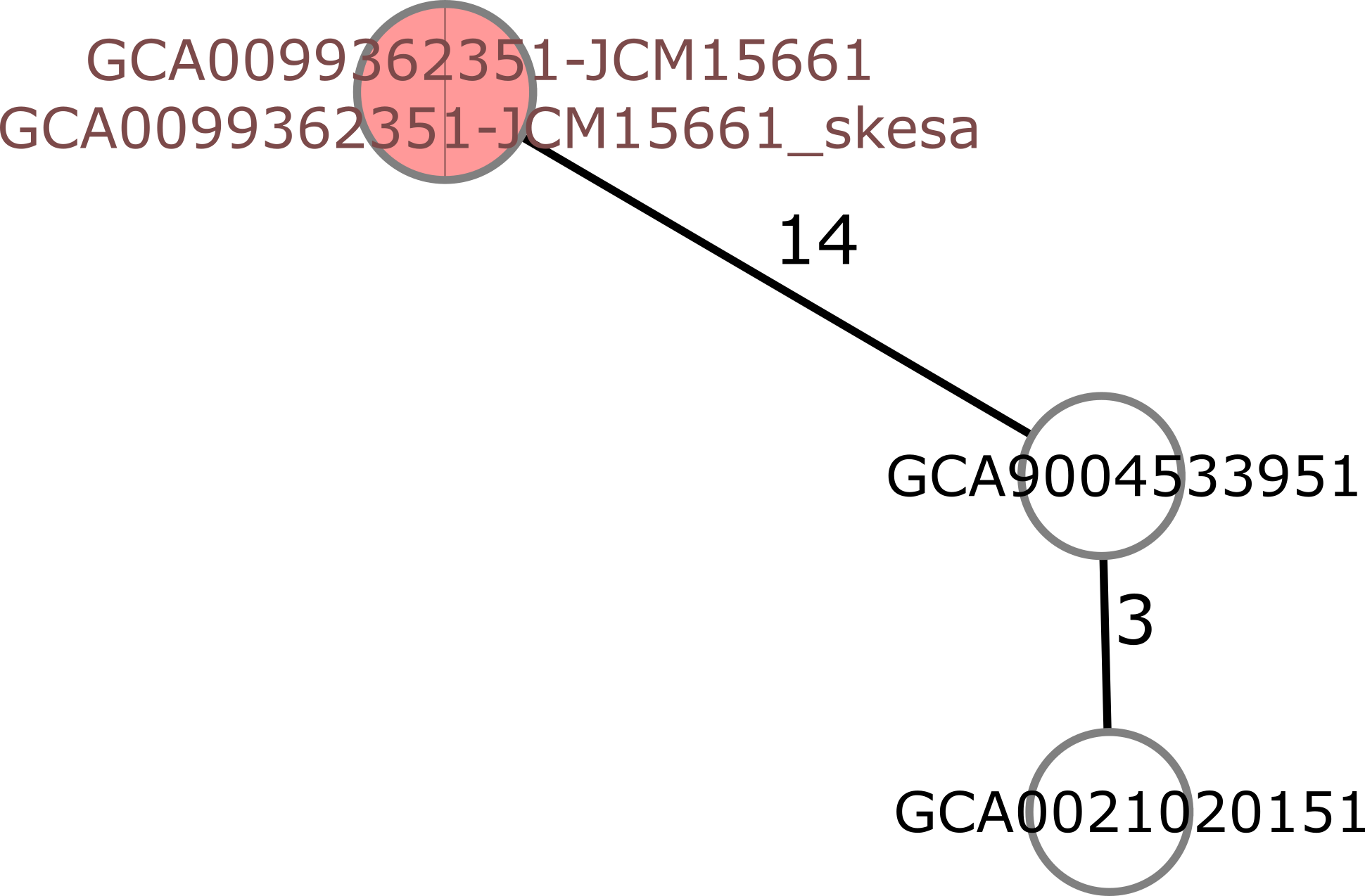


Figure SM2 Minimum spanning tree of three available genomes of the M. xenopi type strain, based on cgMLST analysis of 2565 genes.

### Analysis of sequential isolates from the same patient

For 5 patients, we had sequencing data available for more then one isolate per patient (Table 2).

Table 2 Overview of sequential M. xenopi isolates from 5 patients included in this study

| **NRZ ID** | **Patient ID** | **Collection date** |
| --- | --- | --- |
| 22006825 | P_X_1 | 1999 |
| 22008738 | P_X_1 | 1995 |
| 22006828 | P_X_15 | 2001 |
| 22008741 | P_X_15 | 2001 |
| 22006850 | P_X_54 | 10.07.2009 |
| 22007449 | P_X_54 | 24.07.2008 |
| 22007455 | P_X_54 | 26.01.2015 |
| 22006853 | P_X_60 | 07.03.2012 |
| 22006855 | P_X_60 | 05.09.2012 |
| 22006871 | P_X_82 | 09.10.2018 |
| 22006873 | P_X_82 | 04.11.2019 |

Pairwise comparisons of isolates obtained from the same patient (intrapersonal isolates) all differed by fewer than 10 alleles (max. 6 alleles). Examination of the scatter plot of all pairwise genome distances confirmed this pattern: while the overall allele-distance distribution was broad (0 to >1750 alleles), intrapersonal pairs clustered tightly at the lower end of the range (Figure SM3). However, also a substantial number of pairwise comparisons between isolates from different individuals fell below the 10-allele threshold, suggesting either unrecognized transmission events or contamination or exposure to a common point source.


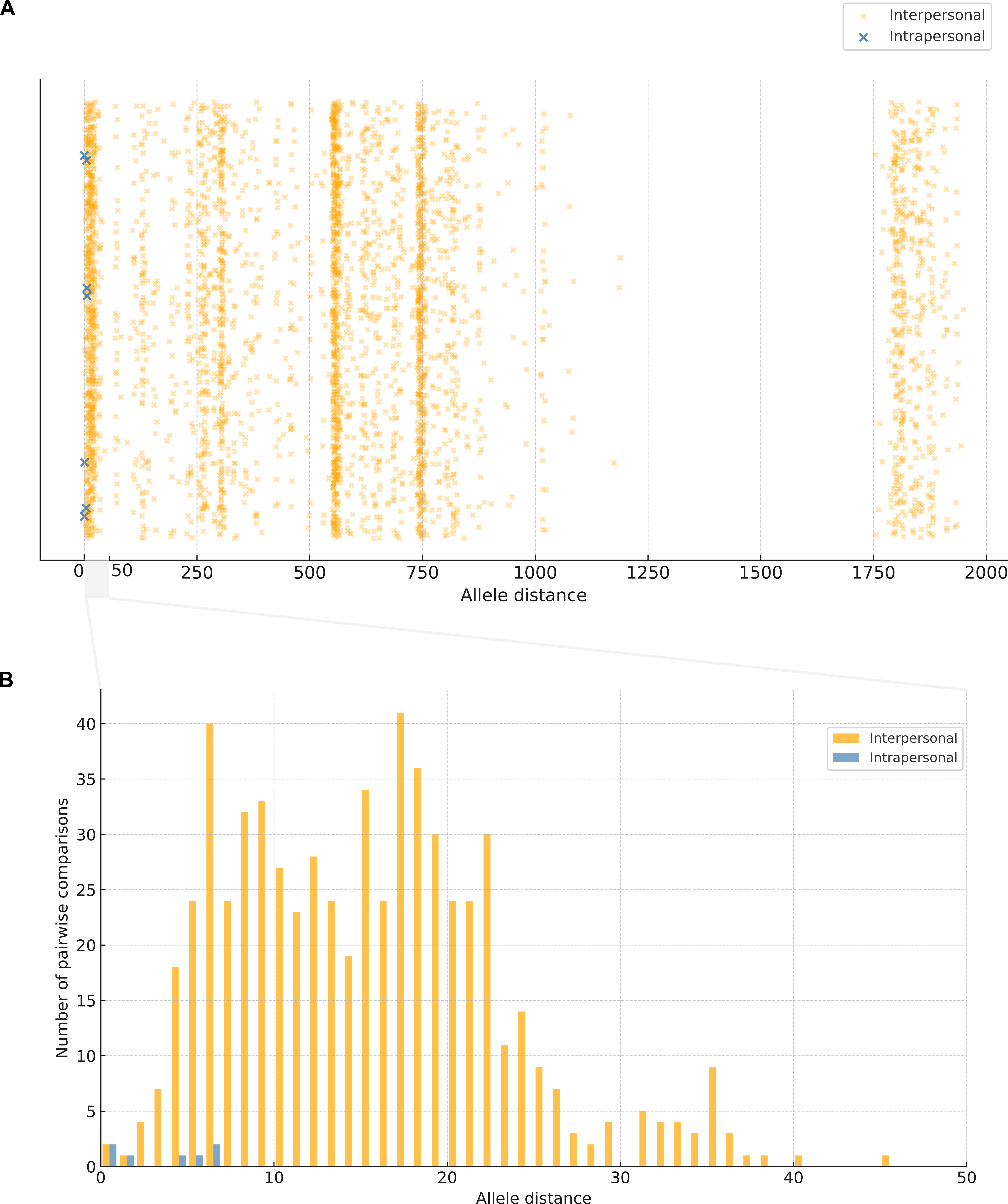


Figure SM3 (A) Scatter plot of all pairwise distances (n=3741 for 87 M. xenopi isolates) calculated using core genome multilocus sequence typing (cgMLST) with 2565 core genes. (B) Allele distance distribution (0-50 alleles) calculated using core genome multilocus sequence typing (cgMLST) with 2565 core genes. Intrapersonal: pairwise comparisons between isolates from same patient. Interpersonal: pairwise comparisons between isolates from different patients.

Based on the analyses of epidemiologically related samples mentioned above, we used a preliminary threshold of 10 alleles to identify putative recent transmission events in M. xenopi (until additional genomic data become available). All pairwise comparisons between isolates from the same patient fell well below this cutoff (maximum: 6 alleles; Figure SM3). We selected a threshold of 10 alleles rather than the observed maximum of 6 because 10 represents a practical and conservative round number that accommodates a small degree of technical variability such as lower sequencing coverage, assembler-related differences (see section 1.6), or uncertain allele calls. Moreover, a threshold around 10 allele differences or single nucleotide polymorphisms (SNP) have been used before in studies of other slow growing mycobacteria such as *M. tuberculosis*^3^ and *M. kansasii*^4^ but also for rapid growers like *M. abscessus*^5^ to define putative recent transmission or short-term epidemiological linkage.

The only epidemiologically related pairwise comparisons exceeding this threshold involved one *M. xenopi* type strain genome that differed by 14 alleles from two other type-strain genomes (Figure SM3), which were themselves closely related (3-allele difference). As this outlier may also reflect the accumulation of mutations over time due to long-term subculturing (the strain was originally isolated in 1959), we did not increase the threshold for recent transmission based on this single discrepancy. Nevertheless, additional data in the future may warrant a revision of the threshold.

Based on the 10-allele threshold, we defined D10 clusters. **D10 clusters** refer to groups (i.e. cluster) of isolates in which each isolate is connected to at least one other member of the cluster by a pairwise allele distance of maximum 10 alleles. This means the cluster is formed using single-linkage: as long as an isolate is within ≤10 alleles of *any* cluster member, it belongs to that cluster. Although clustering is performed using a single-linkage approach with a threshold of 10 alleles (D10), some isolates within the same cluster may exhibit pairwise distances greater than 10 alleles. This is an inherent feature of single-linkage clustering, where membership is based on connectivity rather than on all pairwise distances meeting the threshold.

Please note that allele distances are based on the newly developed cgMLST scheme comprising 2565 loci. Thus, a maximum distance of ‘10 alleles’ indicates that the two isolates differ in the allele assignments of no more than 10 out of the 2,565 core genes.

## Selection of threshold for potential dominant circulating clones

To identify a suitable threshold at higher allelic distances—potentially indicating successful dominant circulating clones (DCCs) —we generated a population-structure curve showing the number of clusters and clustered isolates as a function of the threshold (Figure SM4). This analysis revealed a stable plateau around ~50 alleles, indicating that this cutoff defines robust and reproducible groupings that do not change appreciably with small adjustments of the threshold. In addition, this value corresponded visually to the major groupings (i.e. large, dense clusters similar to *M. abscessus* DCCs) observed in the cgMLST-distance–based neighbor-joining dendrogram (Figure 1, main text).


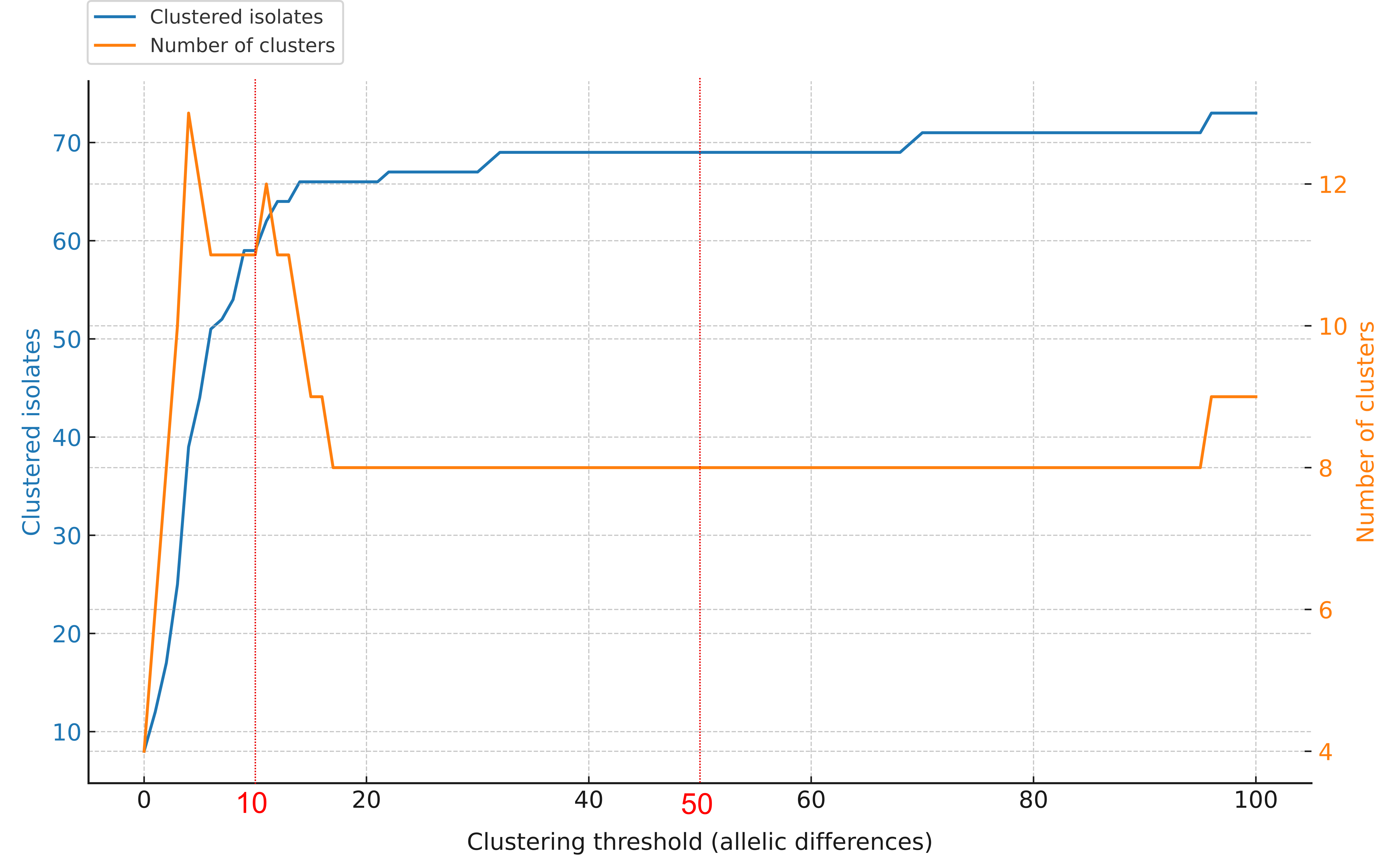


Figure SM4 Clustered isolates and number of clusters in function of the clustering threshold (allelic differences). Chosen thresholds for this study are indicated in red (D10 and D50).

## Effect of assembler and choice of seed genome on cgMLST results

To test the effect of the assembler, we assembled all 87 read sets both with shovill v 1.1.0 using the SKESA 2.4.0 and SPAdes v3.15.0 algorithms. SKESA assemblies consistently had less contigs (between five and 200 less) but also smaller genome lengths (between 30,000 and 160,000 bp less). The median percentage „Good cgMLST targets %“ (i.e. those for which an allele number was assigned, see detailed definition in section 1.3.4) was 99.3% (min. 97.8%, max. 99.8%) for SKESA assemblies and 99.8% (min. 98.2%, max. 100%) for SPAdes assemblies. This indicates that more genes from the cgMLST scheme were either not found in the SKESA assemblies or did not meet the quality criteria according to SeqSphere+ (see section 1.3.3).


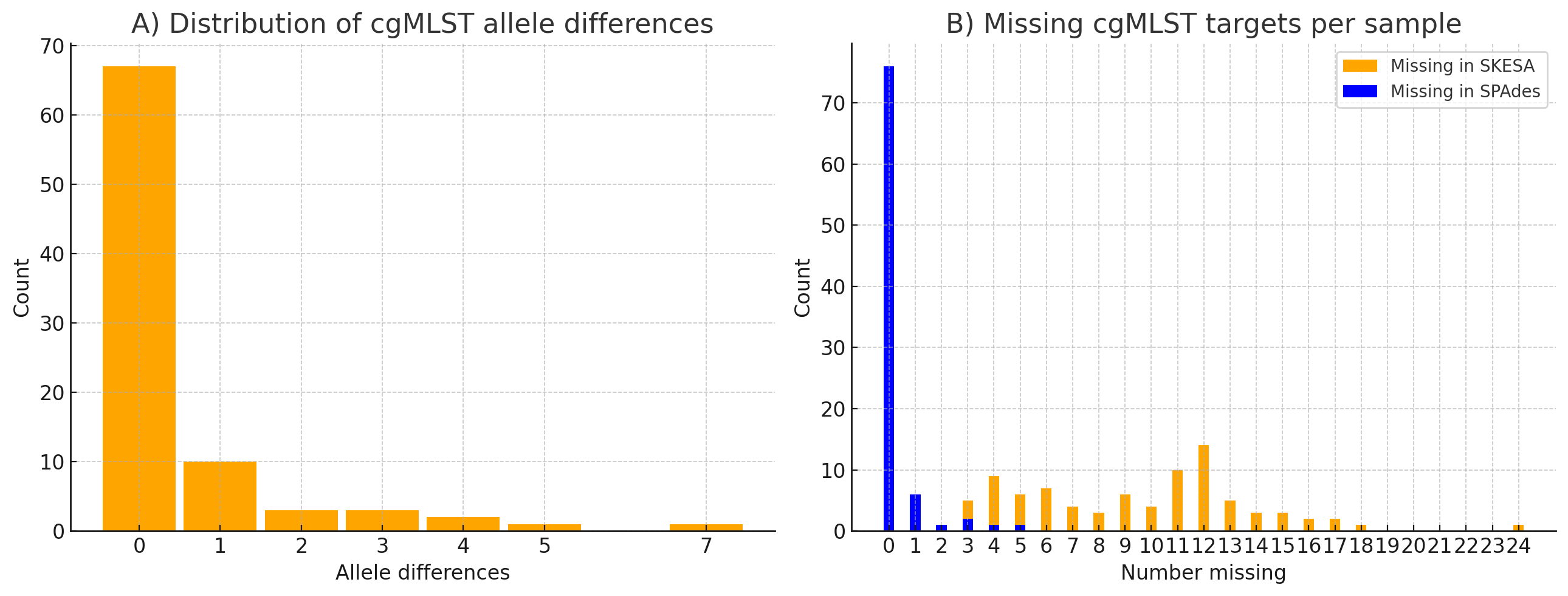


Figure SM5 Effect of assembler on allele calling in 87 M. xenopi isolates. **(A)** Distribution of cgMLST allele differences between SPAdes and SKESA assemblies for the same 87 *M. xenopi* isolates. **(B)** Distribution of the difference in missing cgMLST targets between SPAdes and SKESA assemblies for the same 87 M. xenopi isolates. Count = amount of M. xenopi samples (out of 87).

We found that missing loci were not random in SKESA assemblies. Five targets were missing in more than half of the SKESA assemblies, indicating difficult to assemble genes (Table 3).

Table 3 Top 20 genes of the 2565 cgMLST scheme that were missing in SKESA assemblies.

| **Locus** | **Gene** | **Additional info** | **Missing in X/87 SKESA assemblies** | **% Missing** |
| --- | --- | --- | --- | --- |
| **MYXE_RS01930** | **0** | **peptide ABC transporter substrate-binding protein** | **83** | 95.4 |
| **MYXE_RS10125** | **0** | **WS/DGAT/MGAT family O-acyltransferase** | **80** | 92.0 |
| **MYXE_RS09235** | **sodC** | **superoxide dismutase[Cu-Zn]** | **63** | 72.4 |
| **MYXE_RS24270** | **0** | **zinc finger domain-containing protein** | **58** | 66.7 |
| **MYXE_RS04875** | **0** | **M24 family metallopeptidase** | **53** | 60.9 |
| MYXE_RS05565 | **0** | MogA/MoaB family molybdenum cofactor biosynthesis protein | 47 | 54.0 |
| MYXE_RS04880 | **0** | M24 family metallopeptidase | 47 | 54.0 |
| MYXE_RS00185 | **0** | DUF5631 domain-containing protein | 45 | 51.7 |
| MYXE_RS24765 | **0** | acyl-CoA dehydrogenase family protein | 43 | 49.4 |
| MYXE_RS05570 | **0** | molybdenum cofactor biosynthesis protein MoaE | 42 | 48.3 |
| MYXE_RS24695 | **0** | cupin domain-containing protein | 42 | 48.3 |
| MYXE_RS12720 | **0** | hypothetical protein | 41 | 47.1 |
| MYXE_RS12725 | **0** | MOSC domain-containing protein | 40 | 46.0 |
| MYXE_RS10985 | **0** | quinone-dependent dihydroorotate dehydrogenase | 38 | 43.7 |
| MYXE_RS07665 | **0** | beta-class carbonic anhydrase | 38 | 43.7 |
| MYXE_RS24135 | **0** | hypothetical protein | 37 | 42.5 |
| MYXE_RS12385 | **0** | bifunctional nuclease family protein | 33 | 37.9 |
| MYXE_RS22815 | **0** | thioesterase family protein | 31 | 35.6 |
| MYXE_RS25245 | **0** | Ms4527A family Cys-rich leader peptide | 24 | 27.6 |
| MYXE_RS18205 | **0** | protein export chaperone SatS | 20 | 23.0 |

Note that SPAdes may generate more contiguous assemblies than SKESA (which could explain the higher percentage of good cgMLST targets), although this can occasionally come at the expense of accuracy, potentially resulting in misassembled loci and artificially increased genetic distances. In contrast, SKESA is designed to prioritize accuracy and tends to produce more conservative, but sometimes more fragmented or smaller assemblies excluding problematic regions—particularly GC-rich or repetitive areas— leading to fewer good cgMLST targets. These interpretations remain hypotheses, and additional experiments, including the integration of long-read sequencing, may help clarify the underlying causes.

Importantly, we observed comparable cgMLST distances between sequential isolates from the same patients when genomes assembled with Shovill 1.1.0 using SPAdes v3.15.0 were compared to those assembled with Shovill 1.1.0 using SKESA 2.4.0 (Figure SM6). Therefore, despite the small differences between SKESA and SPAdes assemblies, we feel that both assemblers can be used with the current scheme and threshold of 10 alleles for identification of transmission/outbreak clusters.


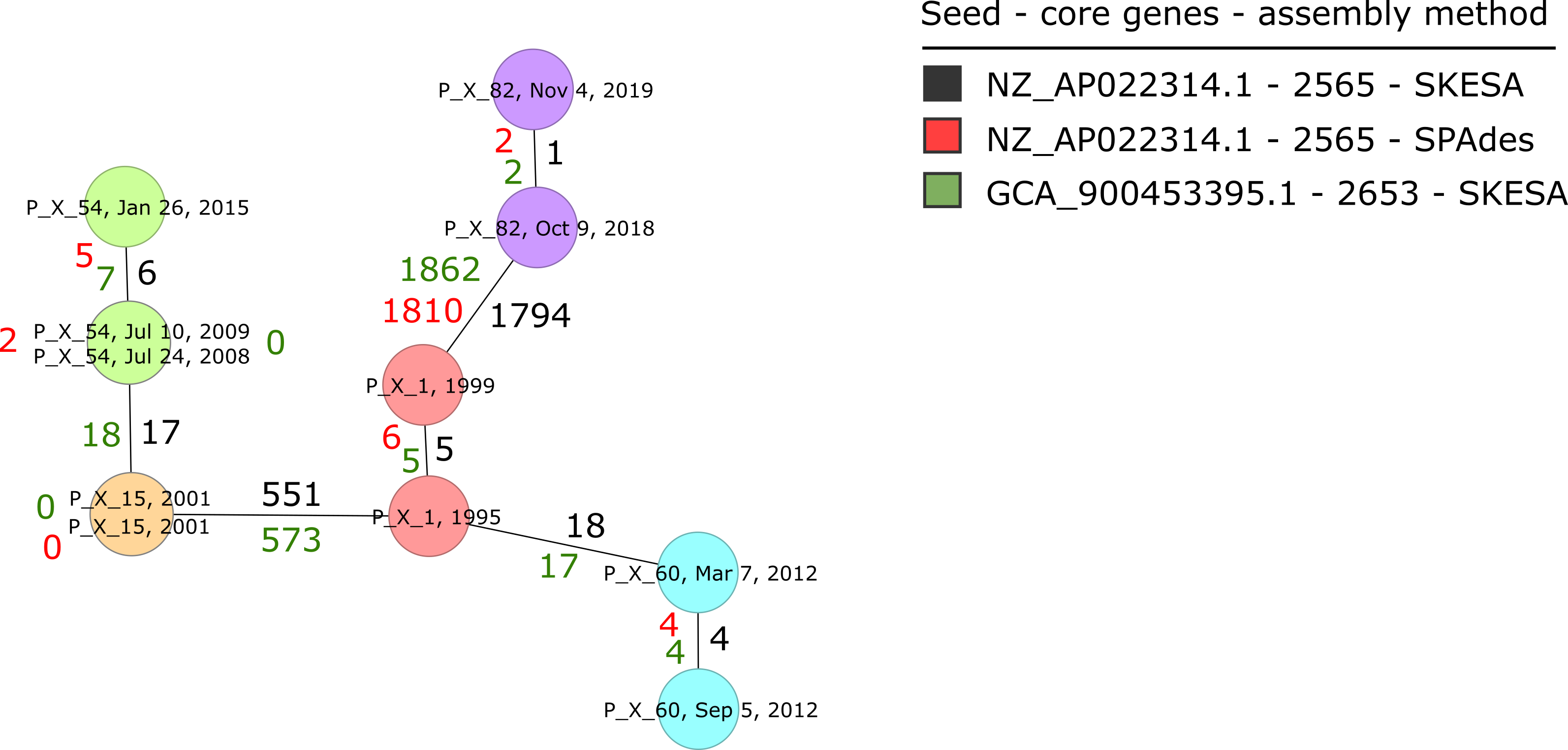


Figure SM6 Minimum spanning tree of sequential *M. xenopi* isolates from five patients. Numbers on the branches represent pairwise allele distances obtained either from genomes assembled with different assemblers (SKESA vs. SPAdes) or from analyses using different cgMLST schemes generated from different seed (reference) genomes (NZ_AP022314.1 vs. GCA_900453395.1). For each patient, a different color was used in the tree.

Analysis of the 87 M. xenopi genomes using an alternative cgMLST scheme generated with a different seed (reference) genome (GCA_900453395.1), while keeping all other parameters identical, produced comparable results despite differences in the total number of core genes (2565 using the complete genome NZ_AP022314.1 versus 2653 using the draft genome GCA_900453395.1). Although allele distances for more distantly related genomes differed modestly between the two cgMLST schemes (up to ~50 alleles), the distances between sequential isolates and the overall population structure remained consistent (Figure SM7 and Figure SM7).


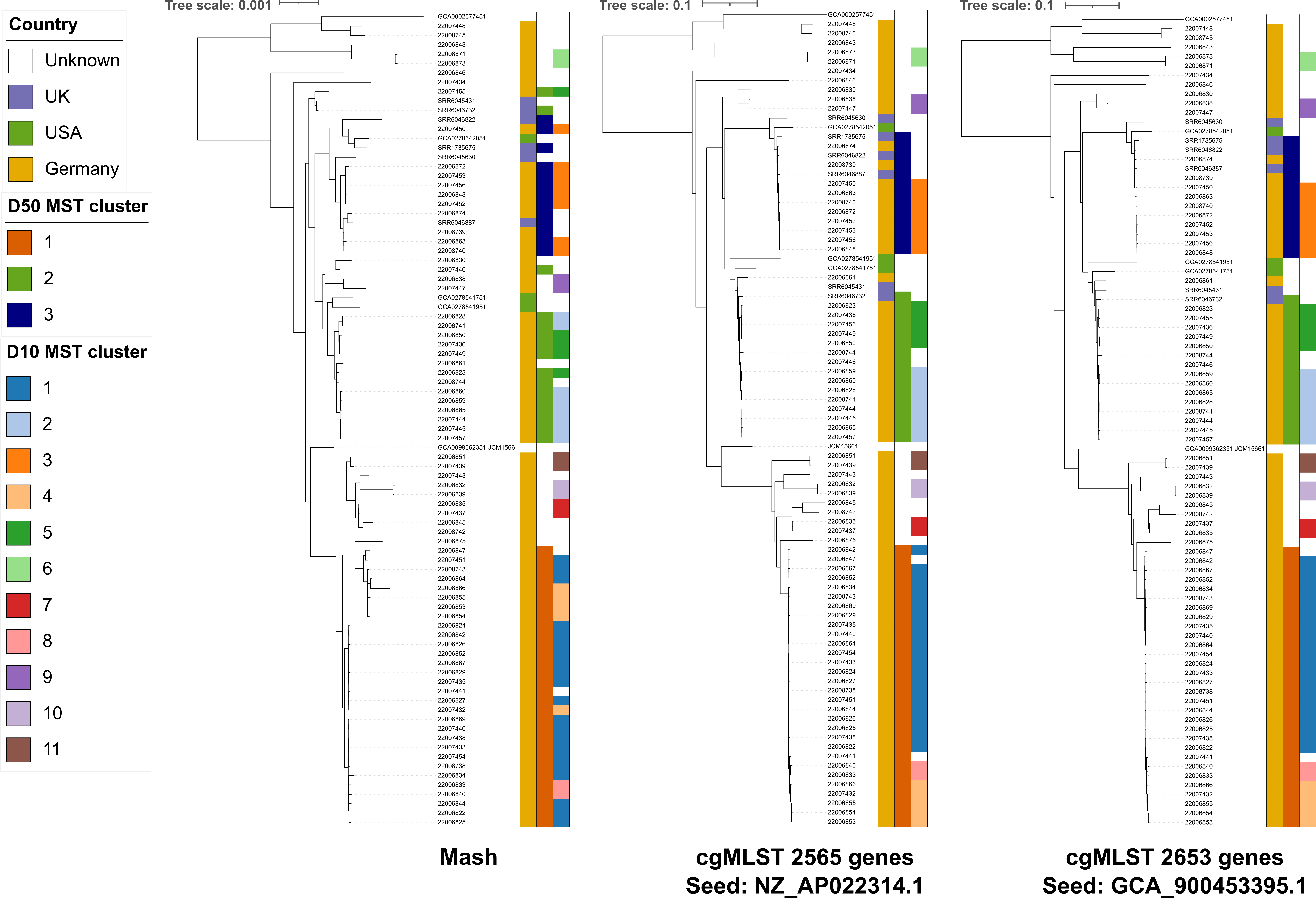


Figure SM7 Comparison of dendrograms generated using different algorithms and different cgMLST schemes. From left to right: a neighbor-joining tree based on Mash distances (calculated with Mashtree); a neighbor-joining tree based on cgMLST distances using a cgMLST scheme comprising 2,565 genes created with NZ_AP022314.1 as the seed genome; and a neighbor-joining tree based on cgMLST distances using a cgMLST scheme comprising 2,653 genes created with GCA_900453395.1 as the seed genome in SeqSphere+. The D10 and D50 clusters correspond to those defined using the final cgMLST scheme comprising 2565 genes applied in this study.

# References

1. Yoshida, M., Fukano, H., Asakura, T., Hisatsune, J. & Hoshino, Y. Complete Genome Sequence of Mycobacterium xenopi JCM15661T, Obtained Using Nanopore and Illumina Sequencing Technologies. *Microbiol. Resour. Announc.* **9**, e01583-19 (2020).

2. Schwabacher, H. A strain of mycobacterium isolated from skin lesions of a cold-blooded animal, Xenopus laevis, and its relation to atypical acid-fast bacilli occurring in man. *Epidemiol. Infect.* **57**, 57–67 (1959).

3. Walker, T. M. *et al.* Whole-genome sequencing to delineate Mycobacterium tuberculosis outbreaks: A retrospective observational study. *Lancet Infect. Dis.* **13**, 137–146 (2013).

4. Machado, E. *et al.* Phylogenomic and genomic analysis reveals unique and shared genetic signatures of Mycobacterium kansasii complex species. *Microb. Genomics* **10**, 001266 (2024).

5. Diricks, M. *et al.* Delineating Mycobacterium abscessus population structure and transmission employing high-resolution core genome multilocus sequence typing. **13**, 4936 (2022).
